# Habitat Redistribution of *Dracaena cochinchinensis* (Lour.) S.C.Chen in Southern China Under Climate and Land-Use Change: Current Distribution, Future Projections, and Conservation Implications

**DOI:** 10.3390/plants15162539

**Published:** 2026-08-21

**Authors:** Zhengnan Zhang, Jie Qiu, Naiwei Li, Linhe Sun, Yajun Chang, Xuan Hu, Kun Dong, Dongrui Yao, Jinfeng Li

**Affiliations:** 1 Institute of Botany, Jiangsu Province and Chinese Academy of Sciences (Nanjing Botanical Garden Mem. Sun Yat-Sen), Nanjing 210014, China; zhangzhengnan_njfu@hotmail.com (Z.Z.); linaiwei_jib@163.com (N.L.); linhesun@cnbg.net (L.S.); changyj@cnbg.net (Y.C.); yaodongrui@cnbg.net (D.Y.); 2 Jiangsu Provincial Key Laboratory for Plant Taxonomy, Resource Conservation and Utilization, Nanjing 210014, China; 3 Jiangsu Engineering Research Center of Aquatic Plant Resources and Water Environment Remediation, Nanjing 210014, China; 4 Nanjing Institute of Environmental Sciences, Ministry of Ecology and Environment, Nanjing 210042, China; qiujie@nies.org; 5 Institute of Agricultural Quality Standards and Testing Technology Research, Hubei Academy of Agricultural Sciences, Wuhan 430064, China; huxuan@hbaas.com; 6 Guangxi Key Laboratory of Environmental Pollution Control Theory and Technology, Guilin University of Technology, Guilin 541006, China; 2020005@glut.edu.cn

**Keywords:** medicinal plant, species distribution, MaxEnt model, climate change, *Dracaena cochinchinensis* (Lour.) S.C.Chen

## Abstract

Climate and land-use change are shifting the spatial distribution and habitat suitability of many plant species, particularly those with narrow ecological niches and limited ranges. *Dracaena cochinchinensis* (Lour.) S.C.Chen is a medicinally important and nationally protected plant species distributed in tropical and subtropical southern China. Predicting its habitat suitability under current and future climate scenarios is essential for understanding its responses to environmental change and guiding conservation and resource management. In this study, an optimized maximum entropy model was used to predict the current habitat suitability of *D. cochinchinensis* in China and to assess changes in habitat suitability under future climate scenarios in the 2090s. The model showed reliable predictive performance for estimating species distribution. Under current conditions, suitable habitats were concentrated in southern China, particularly southern Yunnan, Guangxi, Guangdong, Hainan, Taiwan, and parts of Fujian, covering 23.93 × 10^4^ km^2^ (8.31% of the study area). Mean temperature of the driest quarter (Bio9), temperature annual range (Bio7), and precipitation seasonality (Bio15) were the dominant predictors, highlighting the importance of thermal conditions in shaping species distribution, whereas lithology (Lith) and soil type (ST) were the major non-climatic contributors influencing habitat suitability. Under future scenarios, suitable habitats showed spatial redistribution rather than uniform expansion or contraction. The spatial comparison among future scenarios identified stable habitats in the central and southwestern parts of Hainan Island, south-central Yunnan, and western Guangxi, with habitat losses mainly occurring in coastal South China and limited expansion in southwestern regions. Habitat centroid shifts were scenario dependent, with an eastward shift under SSP1-2.6 and northwestward shifts under SSP3-7.0 and SSP5-8.5, with migration distances ranging from 60.09 to 165.77 km. These results indicate that climate change may substantially reorganize the distribution pattern of *D. cochinchinensis* in southern China. Therefore, future conservation planning should prioritize current high-suitability areas, potential macroclimatically stable areas, and emerging suitable habitats to support long-term preservation and sustainable utilization of nationally protected medicinal species.

## 1. Introduction

*Dracaena cochinchinensis* (Lour.) S.C.Chen is an evergreen arborescent species belonging to the family Asparagaceae and is a characteristic tree-like plant of the northern tropical region. It is distributed in southern Yunnan, Hainan, and southwestern Guangxi Zhuang Autonomous Region of China, as well as in Cambodia, Laos, Thailand, and Vietnam [1,2,3]. The species typically grows on sunny cliffs and rocky mountains, where harsh environmental conditions and highly fragmented habitats restrict population continuity. *D. cochinchinensis* has medicinal, ecological, and ornamental value. Its characteristic deep-red resin, known as Dragon’s Blood (*Resina Draconis*), has been used in traditional Chinese medicine for more than 1500 years [4]. Dragon’s Blood refers to a group of reddish resinous products derived from several plant taxa, with *D. cochinchinensis* as one of the major botanical sources in China [2,5,6,7]. Pharmacological studies have demonstrated multiple bioactivities, including anti-inflammatory, analgesic, antioxidant, antimicrobial, antithrombotic, hemostatic, and wound-healing effects, and it may also promote blood circulation and blood stasis dispersion [8,9,10]. In addition, owing to its evergreen habit and distinctive morphology, *D. cochinchinensis* has ornamental value and is widely used in courtyard landscaping and rocky ecological restoration.

Despite its ecological and economic importance, natural populations of *D. cochinchinensis* have declined substantially in recent decades [11]. The species is characterized by slow growth, poor natural regeneration, and strong habitat specificity. Its natural habitats are mainly restricted to rugged mountains and exposed rocky areas, which further limit population expansion and regeneration [12]. Excessive collection and large-scale exploitation for medicinal use have intensified pressure on wild resources, while deforestation and habitat destruction caused by limestone mining and urban, road, and tourism development further threaten its natural habitats [4,11,13]. Consequently, *D. cochinchinensis* was listed as a rare and endangered plant in the China Plant Red Data Book [14] and was nationally assessed as Vulnerable in China in both 2013 and 2017 [15,16]. At the global level, however, the most recent IUCN Red List assessment, conducted in 2023 and published in 2024, classified *D. cochinchinensis* as Least Concern (LC) [17], replacing its previous Vulnerable status. This updated global assessment indicates a lower estimated extinction risk than previously recognized, but does not necessarily imply a corresponding recovery of wild populations throughout its range. In China, wild populations remain relatively limited and continue to be exposed to pressures from medicinal harvesting and habitat loss or degradation. Despite the updated global assessment, *D. cochinchinensis* remains listed as a Class II nationally protected wild plant in China [18].

Climate change and land use/land cover (LULC) dynamics are key drivers of shifts in species distributions and global biodiversity patterns [19,20,21,22]. Changes in temperature and precipitation can modify habitat suitability and shift distribution ranges, whie LULC changes can directly drive habitat loss and fragmentation, potentially increasing extinction risk, particularly in species with narrow ecological niches, fragmented habitats, and limited dispersal capacity [23,24]. For long-lived, narrowly distributed tree species, the combined effects of climate and LULC shifts may directly affect population persistence, regeneration, and conservation planning. Therefore, assessing how climate change and LULC dynamics influence the habitat suitability of *D. cochinchinensis* is necessary for developing effective conservation and management strategies.

Species distribution models (SDMs), grounded in ecological niche theory, are widely used to predict species’ geographic distributions and habitat suitability under environmental change [25,26]. Among these, the Maximum Entropy (MaxEnt) model is commonly applied because it infers ecological requirements from presence-only occurrence data and environmental variables [23,25,27,28,29,30]. MaxEnt performs reliably even with limited occurrence records, making it suitable for rare and narrowly distributed species [11,20,31]. Previous studies using optimized MaxEnt frameworks have predicted current and future suitable habitats of tree species, identified key climatic constraints, and supported conservation planning, cultivation zoning, and adaptive management under climate change [30,31,32].

However, research on *D. cochinchinensis* has mainly focused on its botanical traits, phytochemical composition, pharmacological activities, medicinal uses, and resource utilization [2,4,10,33]. Quantitative assessments of its geographic distribution and habitat suitability under current and future climate scenarios remain limited. Given its narrow natural range, dependence on rocky habitats, slow growth, poor natural regeneration, and increasing pressure from habitat disturbance and exploitation, it is necessary to identify the key environmental drivers of its distribution and project future habitat shifts. This information is essential for defining priority conservation areas, guiding ex situ conservation and cultivation, and supporting long-term sustainable management of nationally protected medicinal species [25,27,34].

To the best of our knowledge, no study has systematically assessed the potential distribution of *D. cochinchinensis* in China using species distribution modeling under current and future climate scenarios. Consequently, its potential responses to climate change, including shifts in habitat area, spatial distribution, and centroid migration, remain unclear. In this study, an optimized MaxEnt model was used to predict the habitat suitability of *D. cochinchinensis* in China under current climatic conditions and three 2090s climate scenarios (SSP1-2.6, SSP3-7.0, and SSP5-8.5). The objectives were to: (1) identify key environmental factors driving its distribution and predict current habitat suitability; (2) project future suitable habitats and compare changes between current and 2090s conditions; and (3) quantify the magnitude and direction of centroid migration under different scenarios. This study provides a basis for delineating priority conservation areas, supporting ex situ conservation and artificial cultivation, and informing long-term conservation and sustainable management strategies for nationally protected medicinal species.

## 2. Materials and Methods

### 2.1. Study Area

The study area is located in southern China and encompasses 11 provincial-level administrative regions, including Hainan, Guangxi, and Yunnan. It extends from 97.36° to 124.59° E and from 3.83° to 34.32° N, including the terrestrial areas of relevant islands in the South China Sea, with a total land area of approximately 2.12 million km^2^. The region spans tropical and subtropical zones and is characterized by complex topography, including plateaus, mountains, hills, basins, and plains. Elevation generally decreases from the western and southwestern highlands toward the eastern and southeastern coastal areas, resulting in pronounced environmental gradients.

The regional climate is primarily influenced by the East Asian and South Asian monsoons and is generally warm and humid, with abundant and strongly seasonal precipitation. Differences in latitude, elevation, topography, and proximity to the ocean generate substantial spatial heterogeneity in temperature and precipitation. These diverse hydrothermal and topographic conditions support a wide range of vegetation types, including tropical rainforests, tropical monsoon forests, subtropical evergreen broad-leaved forests, montane mixed forests, coniferous forests, and shrublands. Karst landscapes are particularly widespread in southwestern China, where limestone mountains, exposed rocky substrates, and seasonally dry habitats provide distinctive environments for lithophytic plant species. The study area encompasses the major known distribution range of *D. cochinchinensis* in China and its surrounding potentially suitable habitats.

### 2.2. Species Occurrence Records

**Figure 1:**
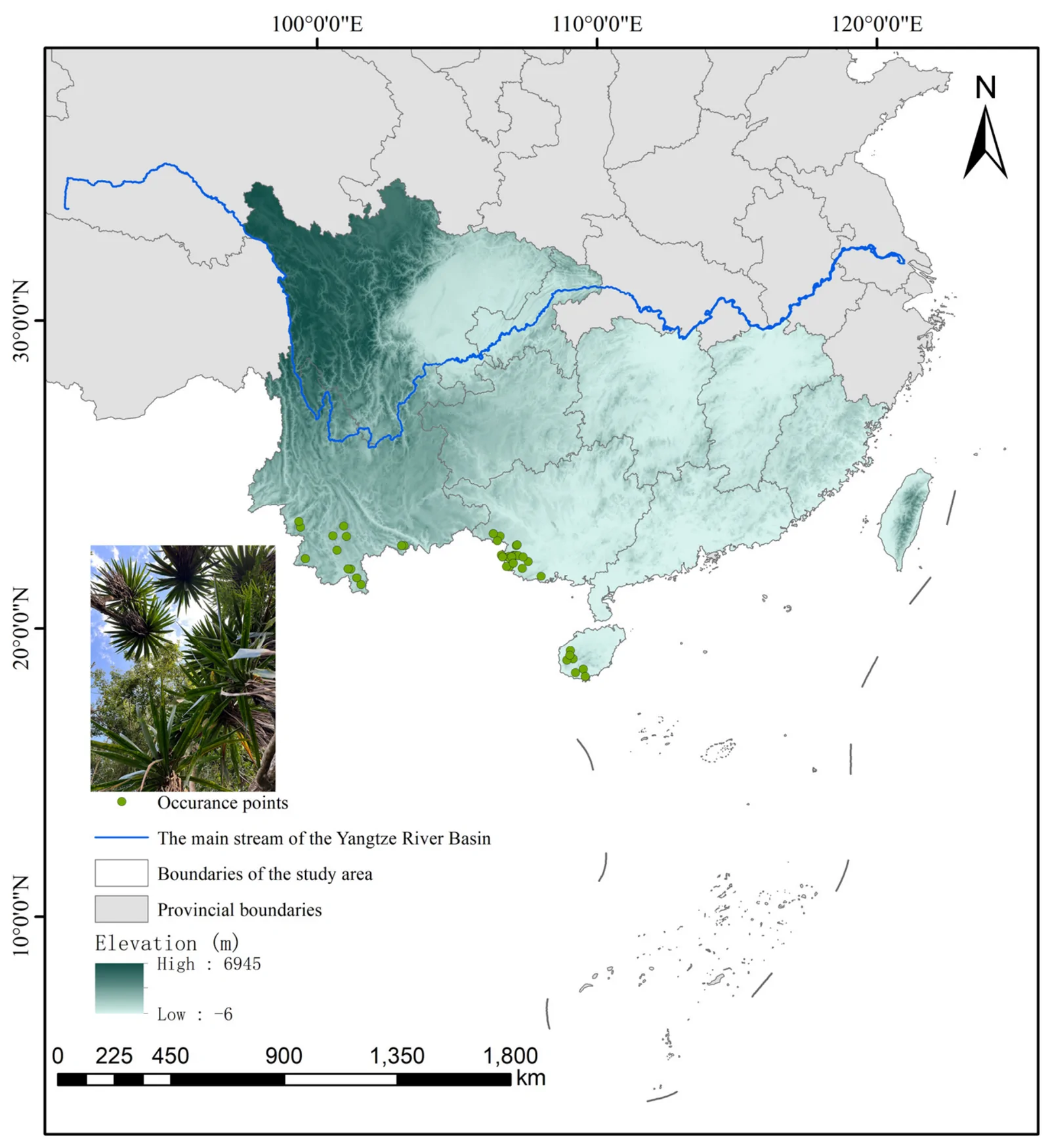
Spatial distributions of occurrence records of *D. cochinchinensis* in southern China. Photograph © Yi-Shan Zhao, licensed under Creative Commons Attribution-NonCommercial 4.0 International (CC BY-NC 4.0), sourced from iNaturalist, Observation ID 196968308. The photograph was taken in Jinggu Dai and Yi Autonomous County, Pu’er City, Yunnan Province, China.

Occurrence records of *D. cochinchinensis* were compiled from multiple sources, including field surveys, related websites, and published literature, Flora of China (FOC), the China Plant Red Data Book [14], the Red Data Book of China Medicinal Plants [35], the Global Biodiversity Information Facility (GBIF; https://www.gbif.org/) (GBIF Occurrence Download. Available online: https://doi.org/10.15468/dL.k42bkq (accessed on 24 November 2024)), the National Specimen Information Infrastructure (NSII), and the Chinese Virtual Herbarium (CVH). All records were standardized and quality-checked, and geographic validation was performed to remove erroneous coordinates, duplicates, incomplete entries, and records from densely urbanized areas or other locations likely associated with artificial cultivation. This process yielded 50 distribution points in China (Figure 1).

To reduce spatial redundancy and sampling bias and limit model overfitting, we used ENMTools software [36] to filter records within the same 30 arc-seconds (~1 km) grid cell, retaining a single occurrence per cell. This resulted in 44 unique records for MaxEnt modeling. The cleaned dataset was formatted as comma-separated values (*.csv) in accordance with the input requirements of MaxEnt v3.4.4 (American Museum of Natural History, New York, NY, USA) [37].

### 2.3. Environmental Variables

We collected 27 environmental variables (Table A1) to characterize the climatic, topographic, edaphic, and land-use conditions potentially influencing the distribution of *D. cochinchinensis*. These variables included 19 bioclimatic variables (Bio1-Bio19), elevation (Elev), northness (Northness), eastness (Eastness), slope (SLP), lithology (Lith), soil type (ST), soil pH in water (T_pH_H2O), and land use/land cover (LULC). The current climatic baseline was defined as 1970–2000 and is hereafter referred to as the current period. Current and future bioclimatic variables were obtained from the WorldClim version 2.1 database (https://worldclim.org) at a spatial resolution of 30 arc-seconds [38]. Aspect was transformed into two continuous components: Northness [cos(Aspect × π/180)] and Eastness [sin(Aspect × π/180)]. Northness reflects the degree to which a slope faces north or south, whereas Eastness reflects the degree to which a slope faces east or west. Both variables range from −1 to 1 [39].

The current climatic scenario and the corresponding habitat-suitability projection were based on climate normals for the baseline period of 1970–2000. Future climatic conditions were represented by the 2090s, corresponding to the mean climate for 2081–2100. Future climate projections were derived from the Beijing Climate Center Climate System Model version 2 Medium Resolution (BCC-CSM2-MR) under the Coupled Model Intercomparison Project Phase 6 (CMIP6) [40]. Three Shared Socioeconomic Pathway (SSP) scenarios were selected: SSP1-2.6, SSP3-7.0, and SSP5-8.5, representing a low-emission sustainable pathway, a regional-rivalry pathway with relatively high emissions, and a fossil-fuel-intensive high-emission pathway, respectively [41,42].

Lithology variable was derived from the Soil and Terrain Database (SOTER) (https://isric.org/explore/soter) (accessed on 24 November 2024) developed by the Food and Agriculture Organization (FAO) and the International Soil Reference and Information Centre (ISRIC), which provides spatially explicit information on lithological and terrain characteristics [43]. Soil variables were obtained from the Harmonized World Soil Database version 1.2 (HWSD v1.2) (https://www.fao.org/soils-portal) (accessed on 24 November 2024), including soil type and soil pH in water for the topsoil layer (0–30 cm) [44]. Topographic variables were derived from the WorldClim v2.1 elevation layer based on the Global Multi-resolution Terrain Elevation Data 2010 (GMTED2010) dataset [38,45]. The current LULC layer was obtained from the European Space Agency Climate Change Initiative Land Cover (ESA-CCI LC) dataset (https://cds.climate.copernicus.eu/datasets/) (accessed on 1 August 2026), which provides consistent global annual land-cover maps derived from satellite observations [46]. The 2000 ESA-CCI land-cover map (version 2.0.7; approximately 300 m resolution) was selected to represent land-cover conditions for the current period. The original ESA-CCI classification follows the FAO Land Cover Classification System (LCCS) and contains 22 basic land-cover classes. To ensure thematic compatibility between current and future LULC predictors, the original ESA-CCI classes were reclassified following the mapping scheme used by Zhang et al. (2023) into the same six land-cover categories used in the future LULC dataset: cropland, forest, grassland, urban, barren land, and water [47]. The harmonized 2000 ESA-CCI layer was subsequently aggregated to the approximately 1 km model grid using the majority class within each target cell. Future LULC layers were obtained from the global 1 km future land use/land cover dataset developed by Zhang et al. (2023) [47]. This dataset provides scenario-specific LULC projections from 2020 to 2100 under representative SSP-RCP scenarios by integrating climate change, socioeconomic factors, land-use demand from the Global Change Analysis Model (GCAM), and the Patch-generating Land Use Simulation (PLUS) model [47]. Accordingly, for each future climate scenario, the corresponding scenario-specific LULC layer was used: SSP1-2.6 climate data were matched with SSP1-2.6 LULC, SSP3-7.0 climate data with SSP3-7.0 LULC, and SSP5-8.5 climate data with SSP5-8.5 LULC.

The MaxEnt model was calibrated using occurrence records and environmental variables for the baseline period (1970–2000) and was then projected to the 2090s under SSP1-2.6, SSP3-7.0, and SSP5-8.5. For future projections, the 19 bioclimatic variables and LULC were updated according to each corresponding SSP scenario, whereas soil and topographic variables were kept constant because they are relatively stable over the study timescale. This approach ensured temporal and scenario consistency and allowed us to evaluate potential shifts in the distribution of *D. cochinchinensis* under the combined effects of future climate and land-use change.

**Figure 2:**
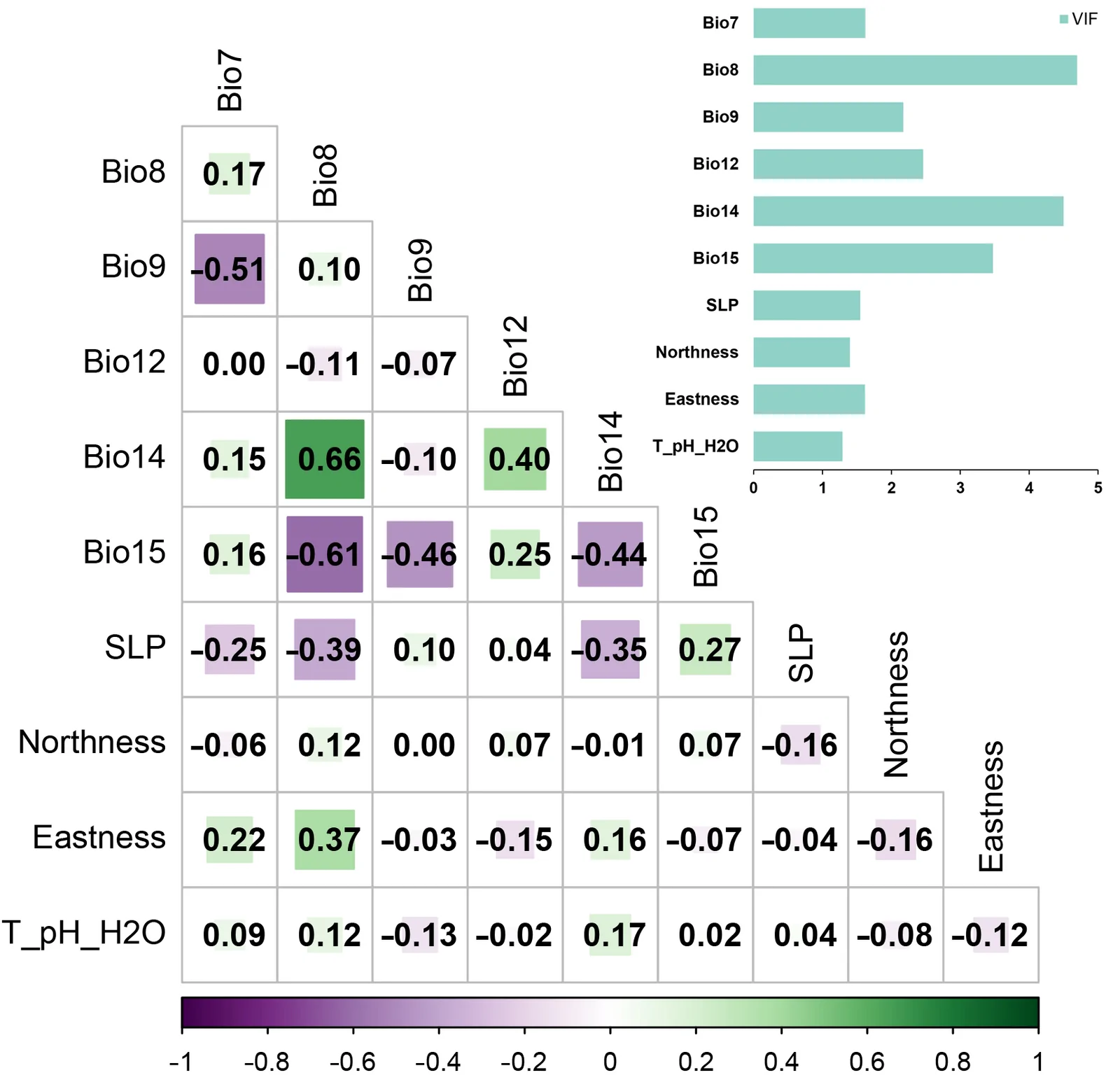
Variable selection based on variance inflation factor (VIF) and Pearson’s correlation analysis.

To ensure consistency with the input requirements of MaxEnt models, all environmental variables were harmonized to a common spatial resolution of approximately 1 km (30 arc-seconds) and the same spatial extent. Continuous predictors were resampled using bilinear interpolation, whereas categorical predictors were processed using the majority class rule where spatial aggregation was required. Lithology: The reclassified 300 m ESA-CCI land-cover layer for 2000 and the lithology layer were aggregated to approximately 1 km by assigning the dominant category within each target grid cell. Other categorical layers, including soil type and future LULC, were already available at approximately 1 km resolution and were directly used after spatial alignment. Because multi-collinearity among environmental variables may reduce model interpretability and affect prediction accuracy [48], we used Variance Inflation Factor (VIF) and Pearson’s correlation coefficient (r) analyses to screen the initial continuous variables. Variables with VIF < 10 and pairwise |r| < 0.8 were retained for subsequent modeling [49,50]. As a result (Figure 2), ten environmental continuous variables were retained after variable screening, including six climatic variables (Bio7, Bio8, Bio9, Bio12, Bio14, and Bio15), three topographic variables (SLP, Northness, and Eastness), and one soil variable (T_pH_H2O). The categorical variables were not subjected to multi-collinearity screening and were directly incorporated into the MaxEnt model as categorical predictors. We conducted variable screening and selection procedures in R version 3.6.1 (R Core Team, Vienna, Austria) within the RStudio environment (RStudio, PBC, Boston, MA, USA).

### 2.4. Species Distribution Modeling

We modeled the current and future habitat suitability of *D. cochinchinensis* using MaxEnt [51]. Species occurrence records and selected environmental variables were used to predict habitat suitability under current conditions and three future climate scenarios for the 2090s (SSP1-2.6, SSP3-7.0, and SSP5-8.5). The model was run with ten cross-validation replicates, a maximum of 500 iterations, a convergence threshold of 0.00001, and 10,000 background points randomly sampled within the accessible area (M), which was defined as a Minimum Convex Polygon (MCP) encompassing all retained occurrence records and expanded outward by a 500 km buffer. The resulting buffered MCP was restricted to terrestrial cells, with marine areas excluded. Model calibration and background sampling were restricted to M, whereas the fitted model was subsequently projected across the entire predefined study area in southern China.

To reduce overfitting and improve model robustness, the regularization multiplier (RM) and feature class (FC) were optimized using the R package ENMeval (version 2.0.5.2). RM values ranged from 0.1 to 4.0 (step 0.1). Six FC combinations were evaluated: L, H, LQ, LQH, LQHP, and LQHPT, where L, Q, P, T, and H denote linear, quadratic, product, threshold, and hinge features, respectively [52]. A total of 240 RM-FC parameter combinations were tested. Model selection was based on AICc, delta.AICc, 10% training omission rate (avg.OR10), and the difference between training and testing area under the receiver operating characteristic curve (AUC) (avg.diff.AUC). The optimal configuration was chosen as the model with the lowest delta.AICc (delta.AICc < 2 considered acceptable), along with lower AICc, avg.OR10, and avg.diff.AUC values. The selected optimal parameters were RM = 3.3 and FC = LQH. Detailed software-configuration parameters are listed in Table S1, and the performance results for candidate models are summarized in Table S2 and Figure S1.

The final MaxEnt model was rerun with optimized parameters, and performance was evaluated using testing AUC, avg.diff.AUC, and the Continuous Boyce Index (CBI) [53]. The testing AUC values range from 0 to 1, with higher values indicating better predictive performance; values >0.9 indicate excellent performance [54,55]. The avg.diff.AUC was used to assess model overfitting, with lower values indicating better model transferability and robustness [56]. The CBI was calculated using the ‘ecospat.boyce()’ function in the R package ecospat. CBI ranges from −1 to +1, with values close to 1 indicating high agreement between observed and predicted habitat suitability, whereas values ≤ 0 indicate performance no better than random prediction [53]. A jackknife test was conducted to estimate percent contribution and permutation importance of environmental variables, identifying the main factors shaping the habitat suitability of *D. cochinchinensis*.

### 2.5. Habitat Suitability

After identifying potentially suitable habitat areas, spatial analysis in ArcGIS 10.5 (Esri, Redlands, CA, USA) was used to classify habitat suitability values. Following previous MaxEnt-based studies [57,58,59], we applied the maximum training sensitivity plus specificity logistic threshold (0.305) as the objective cutoff to distinguish unsuitable from suitable habitats. This threshold has been widely recommended for converting continuous suitability outputs into binary presence–absence maps due to its balanced omission and commission error rates. To further characterize habitat suitability gradients, the values above the threshold were subdivided into three equal-interval classes, resulting in four categories: unsuitable (0–0.305), low suitability (0.305–0.536), moderate suitability (0.536–0.768), and high suitability (0.768–1.000).

### 2.6. Area Changes and Centroid Migration of Habitat Suitability

Changes in suitable habitat area were quantified, and centroid shifts between current and future projected distributions were analyzed using SDMtoolbox v1.0b [60]. SDMtoolbox is a Python 2.7-based GIS toolkit that summarizes species range shifts by reducing suitable habitat to a centroid point and generating vectors representing the magnitude and direction of movement over time [60]. To identify cross-scenario consensus in habitat transitions relative to the current period, the change maps for SSP1-2.6, SSP3-7.0, and SSP5-8.5 were overlaid on a cell-by-cell basis. Cells were classified as consensus stable, expanded, or loss habitat only when all three scenarios showed the same transition type, whereas cells with inconsistent responses among scenarios were classified as mixed/scenario-dependent areas. All spatial calculations were conducted in the Asia North Equidistant Conic coordinate system. Centroid migration of *D. cochinchinensis* was tracked between the current period and the 2090s under different climate scenarios to assess potential distributional shifts.

## 3. Results

### 3.1. Environmental Variable Importance and Response Curves

**Figure 3:**
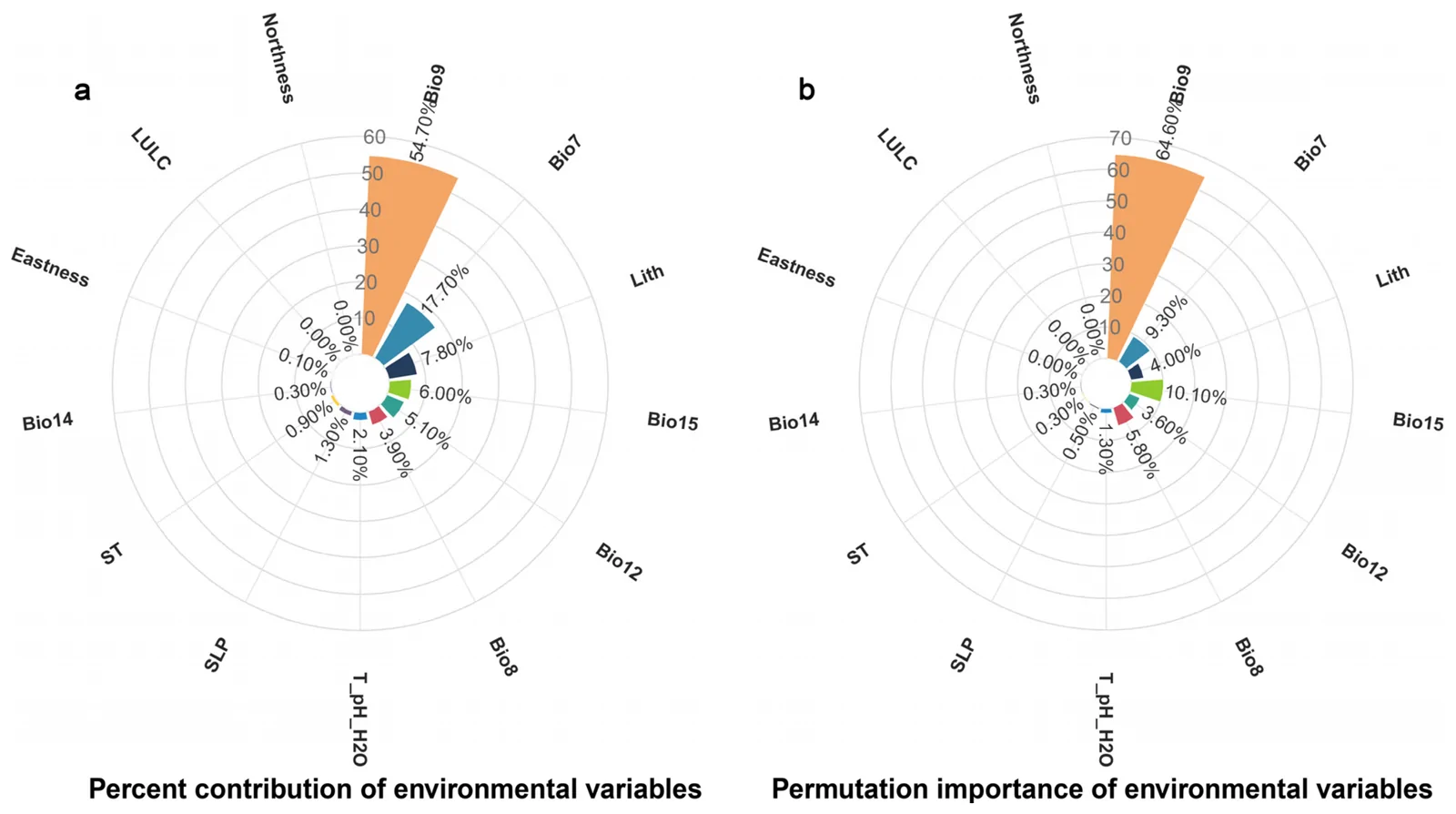
Contribution of environmental variables to the habitat suitability of *D. cochinchinensis*. (**a**) Percent contribution of each environmental variable in the MaxEnt model. (**b**) Permutation importance of environmental variables. Higher values indicate greater influence on model performance.

The contribution analysis indicated that climatic variables were the dominant model predictors of the habitat suitability of *D. cochinchinensis* (Figure 3). Among all environmental predictors, Bio9 (mean temperature of the driest quarter) showed the highest percent contribution (54.70%), followed by Bio7 (annual temperature range, 17.70%), Lith (7.80%), Bio15 (precipitation seasonality, 6.00%), and Bio12 (annual precipitation, 5.10%) (Figure 3a). Bio8 (mean temperature of the wettest quarter) contributed 3.90%, whereas T_pH_H2O (2.10%), SLP (1.30%), ST (0.90%), Bio14 (0.30%), Eastness (0.10%), LULC (0.00%), and Northness (0.00%) showed relatively minor contributions. Permutation importance analysis further identified Bio9 as the most influential variable, accounting for 64.60% of total permutation importance, indicating high model sensitivity to this predictor (Figure 3b). Bio15 (10.10%), Bio7 (9.30%), Bio15 (5.80%), Lith (4.00%) and Bio12 (3.60%)also contributed substantially, whereas the remaining variables had relatively minor effects (<1.30%).

**Figure 4:**
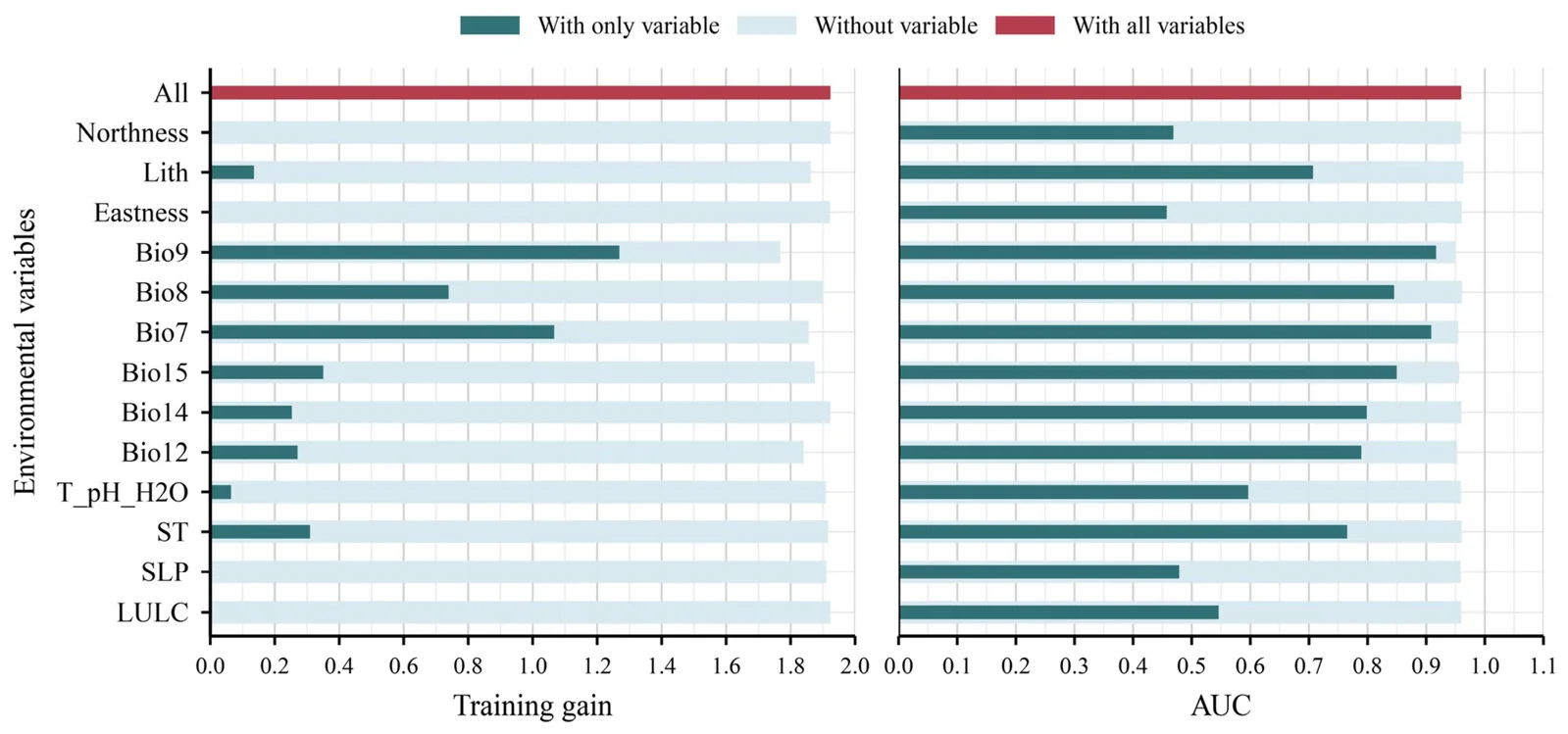
Jackknife test of environmental variable importance in the MaxEnt model for *D. cochinchinensis*. Environmental variable importance was assessed using training gain and AUC. Dark green bars represent models using each variable in isolation, light blue bars represent models excluding each variable, and red bars represent models using all variables.

The jackknife test showed that Bio9 produced the highest training gain and AUC when used alone, followed by Bio7 in both metrics (Figure 4). Bio8 ranked third in training gain, whereas Bio15 ranked third in AUC, indicating that these temperature-related variables provided the most informative independent predictors of species distribution. In contrast, Northness, Eastness, and SLP produced extremely low training gains when used alone, while Eastness, Northness, and SLP had the lowest individual AUC values, suggesting limited independent predictive power. In addition, Lith and ST achieved relatively high training gains and AUC when used individually, ranking as the top two non-climatic predictors. This suggests that geological and edaphic factors also play an important role in shaping habitat suitability, although their explanatory power was lower than that of the dominant climatic variables.

Overall, all climate-related variables exhibited comparatively high AUC values when used in isolation, further confirming their strong predictive ability. Notably, Lith and ST were the leading non-climatic variables in terms of AUC, highlighting their secondary but non-negligible contribution to the model performance. Model performance remained largely unchanged when individual variables were omitted, indicating overlap in environmental information among predictors. Overall, Bio9, Bio7, and Bio 15 were the strongest independent climatic predictors, further highlighting the dominant role of climatic conditions in shaping the habitat suitability of *D. cochinchinensis*.

**Figure 5:**
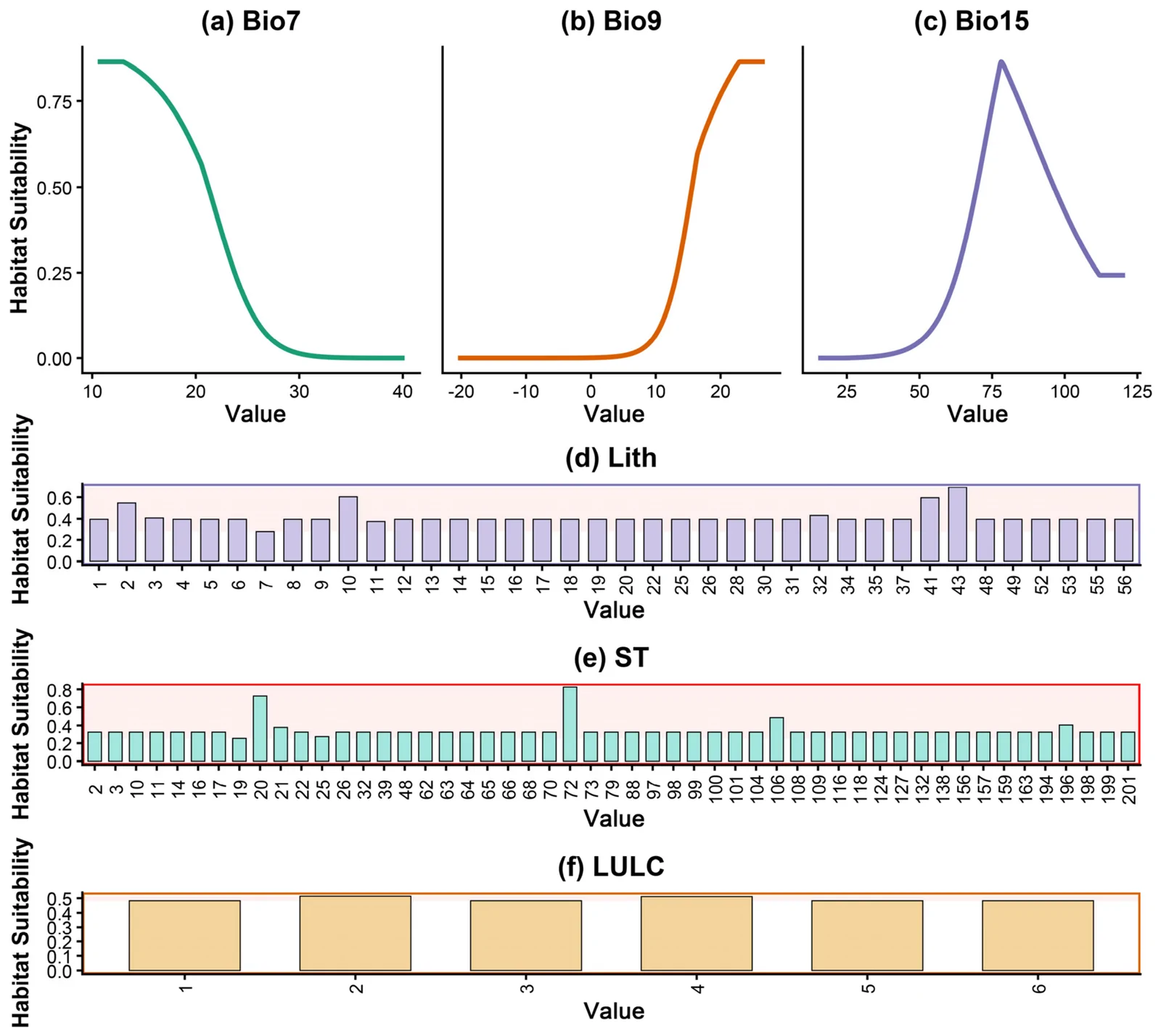
Response curves of dominant environmental variables affecting habitat suitability of *D. cochinchinensis* in the MaxEnt model. (**a**) Bio7: temperature annual range; (**b**) Bio8: mean temperature of the wettest quarter; (**c**) Bio9: mean temperature of the driest quarter; (**d**) Lith, lithology; (**e**) ST, soil type; and (**f**) LULC, land use/land cover. The *y*-axis represents the predicted habitat suitability. Definitions of the categorical codes for Lith, ST, and LULC are provided in Supplementary Table S3.

Response curves showed that habitat suitability was strongly influenced by temperature-related variables Bio7, Bio9, and Bio15, as well as Lith and ST, whereas responses to LULC were comparatively limited (Figure 5). Suitability exhibited a nonlinear response to Bio7, remaining relatively high when the annual temperature range was below approximately 12.96 °C, but declining sharply beyond this threshold (Figure 5a). This pattern suggests that *D. cochinchinensis* was associated with higher predicted habitat suitability under relatively smaller annual temperature ranges. For Bio15, it exhibited a unimodal response, remaining low at lower levels of precipitation seasonality but increasing rapidly when Bio15 exceeded approximately 55.00. Suitability reached its maximum at approximately 78.1 and then gradually declined with further increases in precipitation seasonality, eventually stabilizing at a relatively low level above approximately 112.00 (Figure 5b). Similarly, suitability began to increase markedly when the mean temperature of the driest quarter (Bio9) exceeded approximately 12.00 °C, with the most rapid increase occurring around 15.73 °C. Suitability subsequently increased more gradually and approached a stable high level above approximately 22.95 °C (Figure 5c). These response patterns indicate that relatively smaller annual temperature ranges, intermediate levels of precipitation seasonality, and warmer conditions during the driest quarter were associated with higher predicted habitat suitability of *D. cochinchinensis*. The highest suitability values were observed for lithological class 43 (Slate, phyllite (pelitic rocks)/Schist), followed by class 10 (Limestone and other carbonate rocks), class 41 (Bare rock/rock outcrop), and class 2 (Diorite). Habitat suitability also varied substantially among soil types (Figure 5e). For ST, the highest suitability values were associated with Haplic Ferralsols (ST = 72), Ferric Acrisols (ST = 20), while Haplic Luvisols (ST = 106; 0.488) and bare rock/rock outcrop (ST = 196; 0.405) also showed relatively higher values than most other soil categories. Most remaining soil types exhibited similar predicted suitability values of approximately 0.33. In contrast, predicted suitability varied only slightly among the six LULC classes (Figure 5f); class 2 (Forests; 0.514) showed relatively higher values than the other classes.

### 3.2. Model Performance and Validation

**Figure 6:**
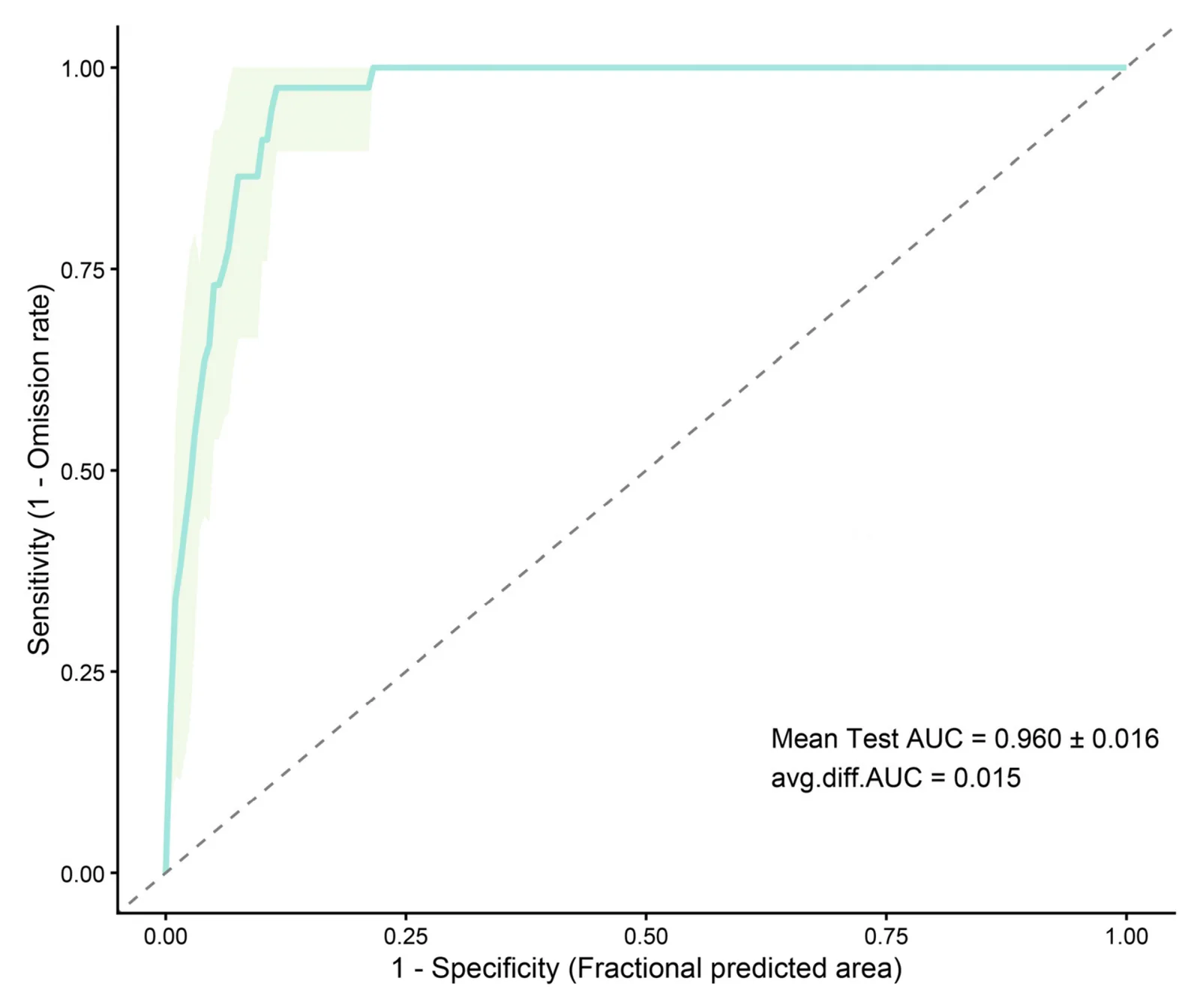
Receiver operating characteristic (ROC) curve and area under the curve (AUC) of the optimized MaxEnt model for *D. cochinchinensis*. The solid cyan line represents the mean ROC curve, and the light-green shaded area indicates the variation among model replicates. The gray dashed diagonal line represents the no-discrimination reference.

**Figure 7:**
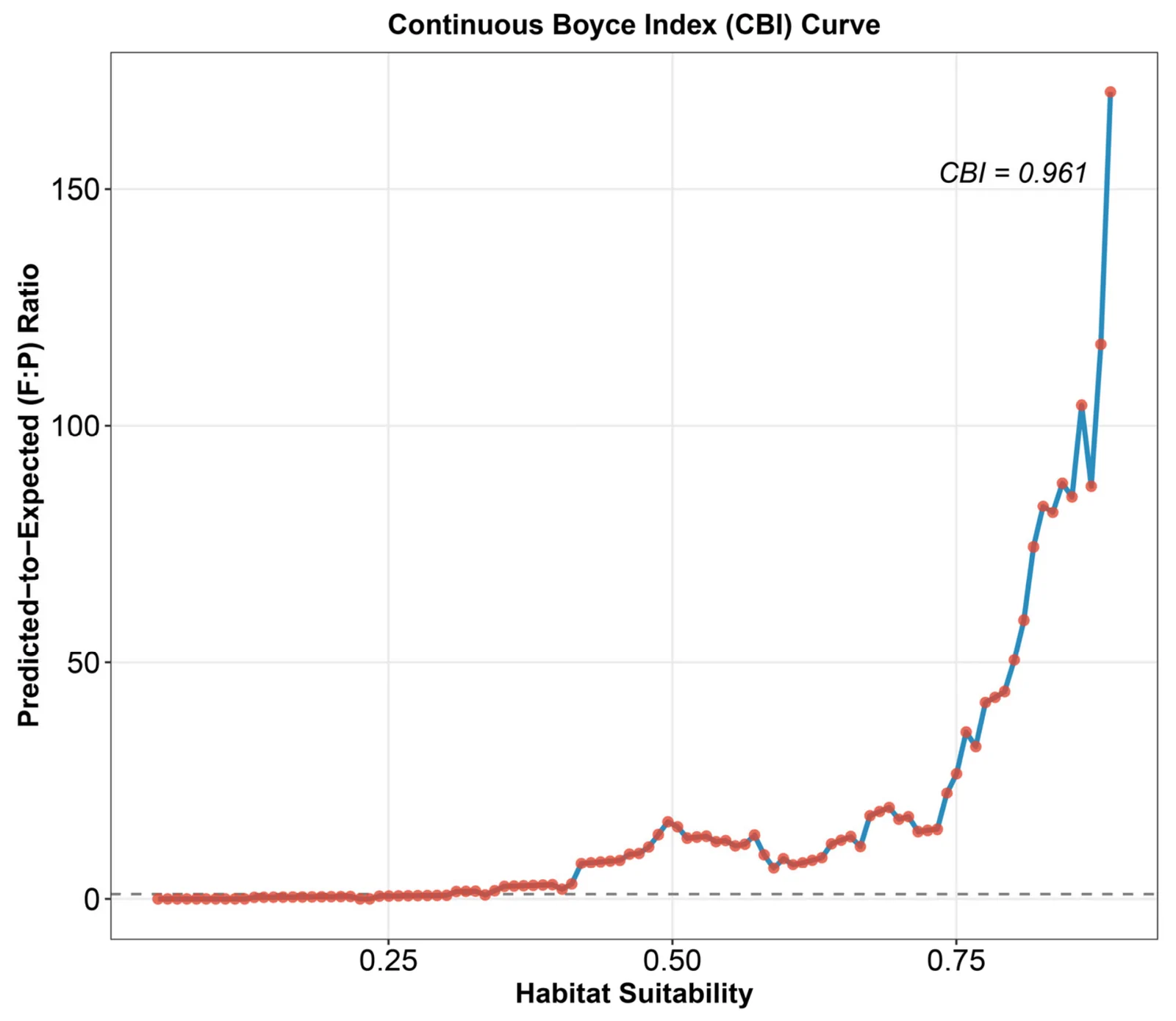
Continuous Boyce Index (CBI) evaluation of the MaxEnt model for *D. cochinchinensis*. The CBI was calculated using the continuous moving-window approach implemented in ecospat.boyce (nclass = 0, default window width, res = 100). The curve represents the predicted-to-expected (P/E) ratio across the continuous habitat-suitability gradient. The gray dashed horizontal line at P/E = 1 represents the random-expectation reference, where the predicted frequency equals the expected frequency.The distribution of validation occurrence records across predicted suitability values is shown along the *x*-axis to indicate sample support across the suitability gradient.

The predictive performance of the optimized MaxEnt model was evaluated using the receiver operating characteristic (ROC) curve and the area under the curve (AUC). The model achieved a mean AUC of 0.960 ± 0.016 (mean ± SD) across replicate runs (Figure 6), indicating excellent predictive accuracy. The average difference between the training and test AUC values (avg.diff.AUC) was 0.015, suggesting limited model overfitting and good generalization ability. In addition, the continuous Boyce index further confirmed the reliability of the model, with a CBI value of 0.961 (Figure 7). The Boyce curve showed a clear increase in the predicted-to-expected (F:P) ratio with increasing habitat suitability, indicating that occurrence records were concentrated in areas assigned high suitability scores.

### 3.3. Current Habitat Suitability of D. cochinchinensis

**Figure 8:**
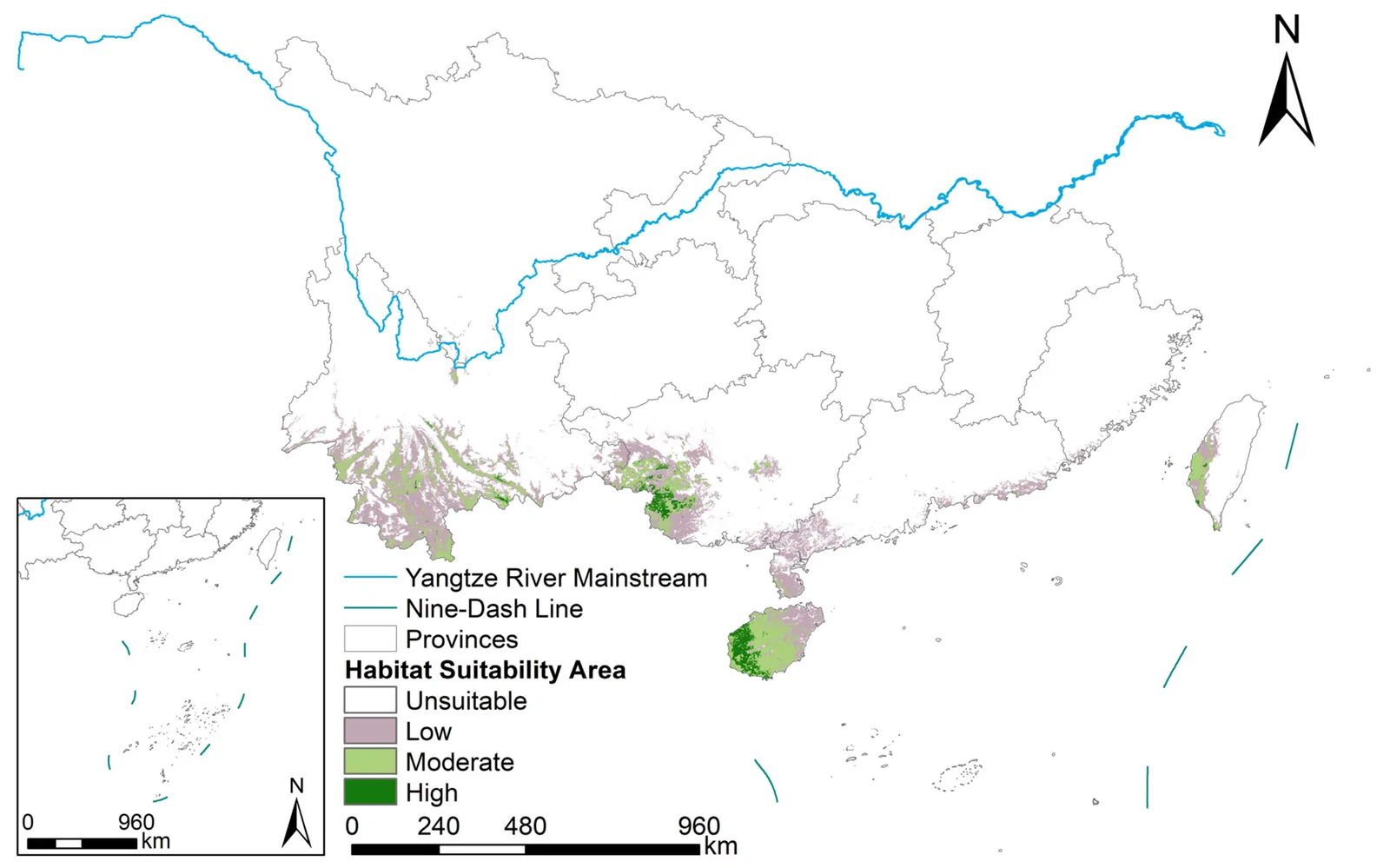
Current habitat suitability of *D. cochinchinensis* in southern China.

The current suitable habitat area of *D. cochinchinensis* was concentrated in southern China, particularly in tropical and subtropical regions south of the Yangtze River (Figure 8). Suitable habitats were discontinuously distributed across southwestern and southeastern China, with major suitable areas located in southern Yunnan, Guangxi, Guangdong, Hainan, and Taiwan, with scattered suitable habitats in parts of Fujian. In contrast, northern China and most areas north of the Yangtze River were predicted to be unsuitable.

Under current climatic conditions, the total suitable habitat area was 23.93 × 10^4^ km^2^, representing 8.31% of the study area. Low-, moderate-, and high-suitability habitats occupied 15.51 × 10^4^ km^2^ (5.38%), 7.09 × 10^4^ km^2^ (2.46%), and 1.33 × 10^4^ km^2^ (0.46%), respectively. Highly suitable habitats were primarily concentrated on Hainan Island, in southern Yunnan, and in localized areas of Guangxi and Taiwan, whereas low- and moderate-suitability habitats formed a broader belt across southern China.

### 3.4. Future Habitat Suitability of D. cochinchinensis

**Figure 9:**
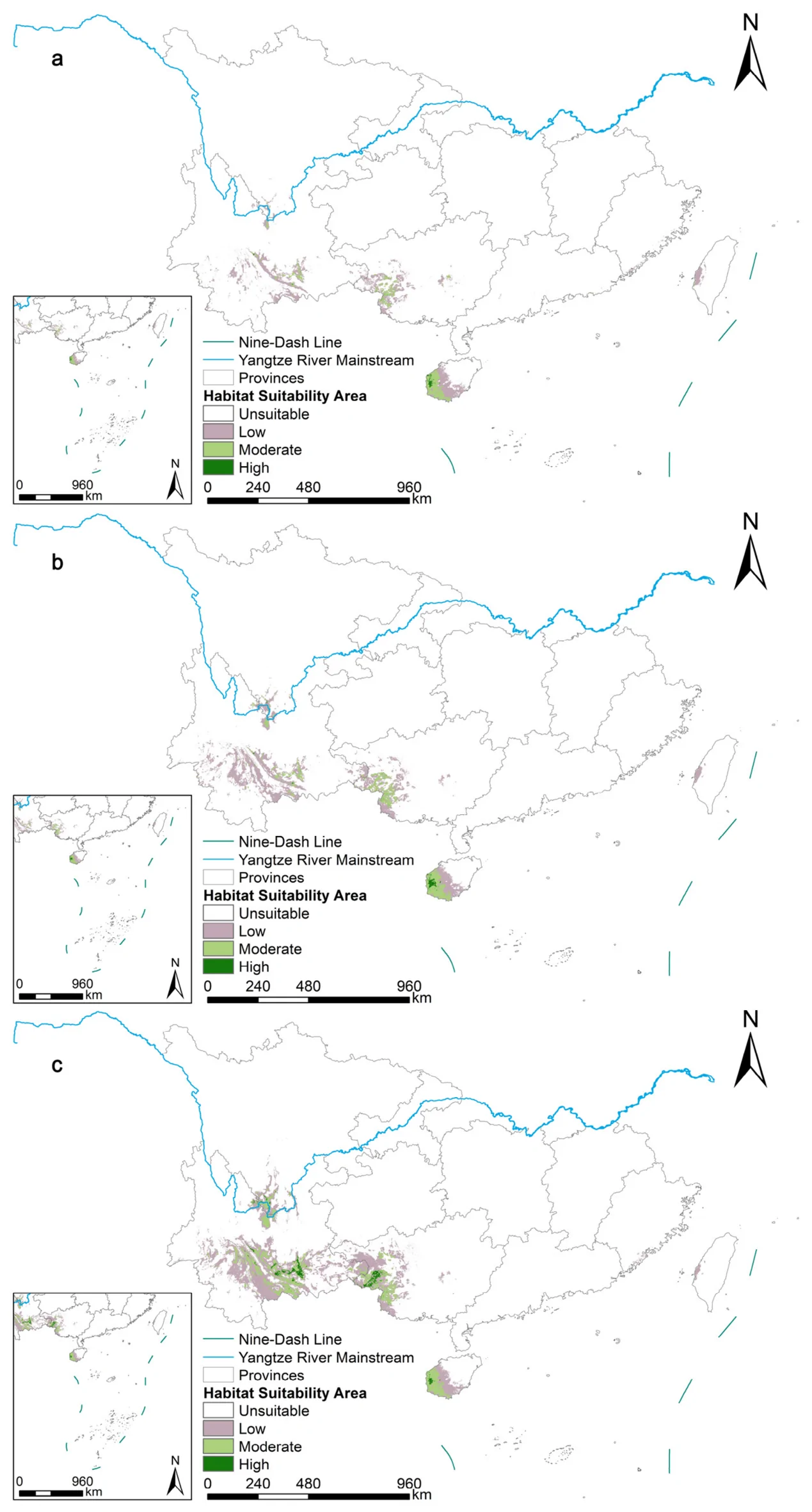
Future habitat suitability of *D. cochinchinensis* under different climate scenarios in the 2090s: (**a**) SSP1-2.6, (**b**) SSP3-7.0, and (**c**) SSP5-8.5.

The extent of suitable habitat varied considerably among future climate scenarios (Figure 9). Under SSP1-2.6 in the 2090s, the total suitable habitat area was projected to be 7.23 × 10^4^ km^2^, including 5.45 × 10^4^ km^2^ of low-, 1.69 × 10^4^ km^2^ of moderate-, and 0.09 × 10^4^ km^2^ of high-suitability habitat. Under SSP3-7.0, the total suitable habitat area increased to 10.70 × 10^4^ km^2^, comprising 8.01 × 10^4^ km^2^ of low-, 2.53 × 10^4^ km^2^ of moderate-, and 0.17 × 10^4^ km^2^ of high-suitability habitat. The largest suitable habitat area was projected under SSP5-8.5, reaching 18.82 × 10^4^ km^2^, with 12.96 × 10^4^ km^2^, 5.15 × 10^4^ km^2^, and 0.70 × 10^4^ km^2^ classified as low-, moderate-, and high-suitability habitats, respectively.

### 3.5. Changes in Suitable Habitat Area and Centroid Shifts

**Figure 10:**
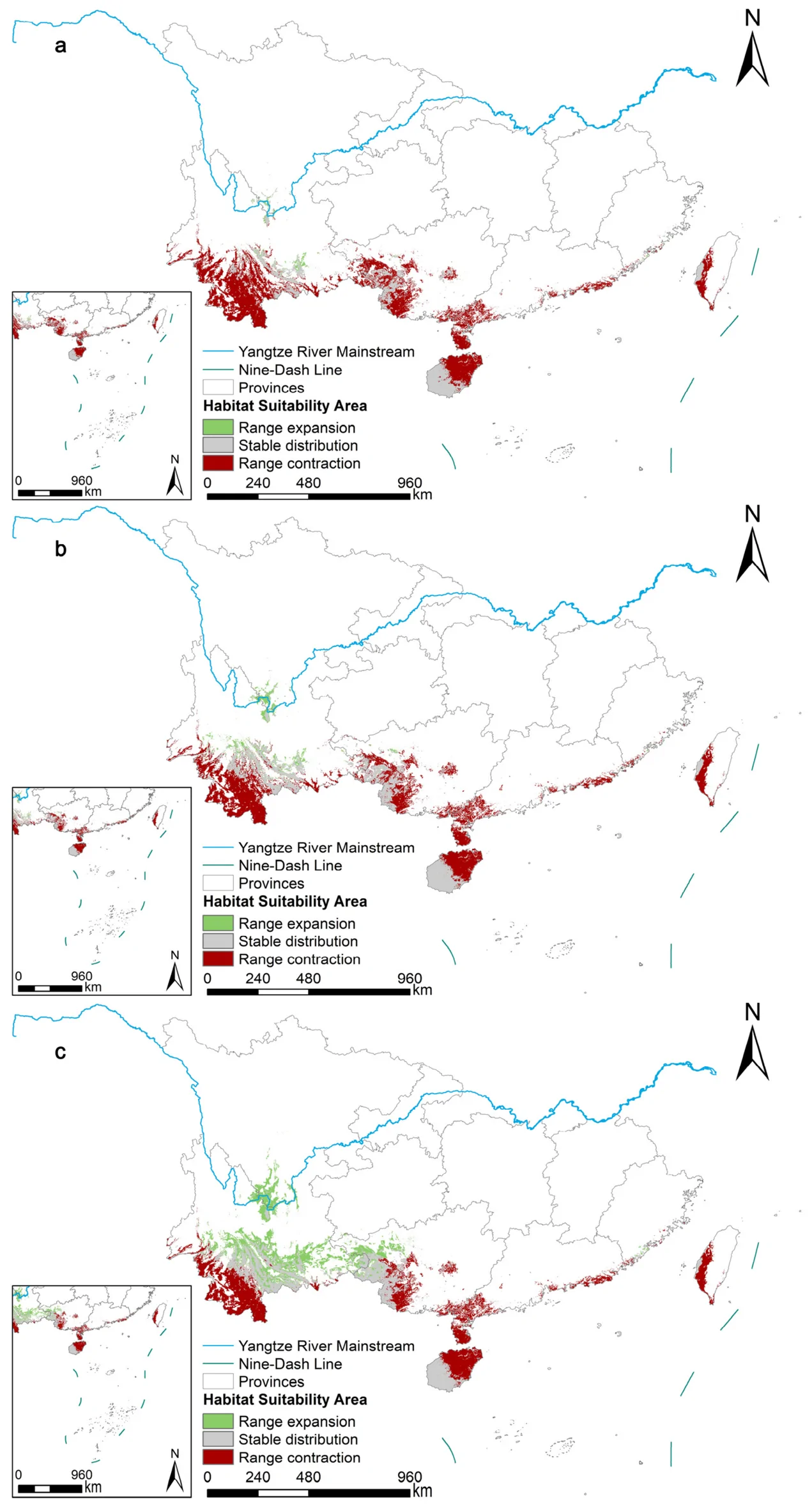
Predicted changes in suitable habitat distribution of *D. cochinchinensis* under future climate scenarios in southern China: (**a**) SSP1-2.6, (**b**) SSP3-7.0, and (**c**) SSP5-8.5. Green areas indicate habitat expansion, gray areas indicate stable suitable habitats, and red areas indicate habitat contraction relative to the current distribution.

**Figure 11:**
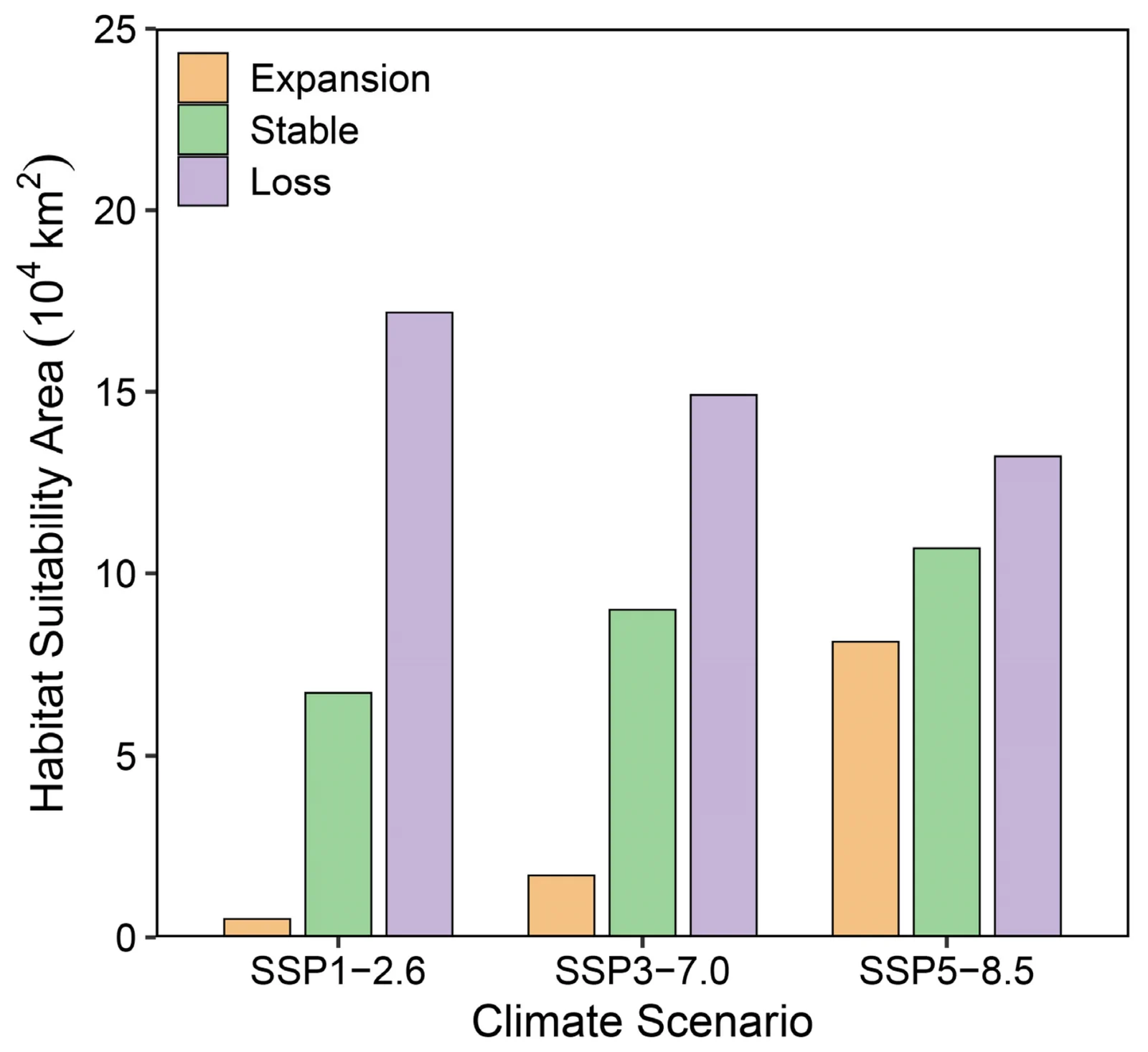
Suitable habitat transitions of suitable habitat area of *D. cochinchinensis* under different climate scenarios (2090s) relative to the current scenarios.

Habitat transition analysis revealed distinct differences among future climate scenarios (Figure 10 and Figure 11). Under SSP1-2.6, 6.72 × 10^4^ km^2^ of suitable habitat remained stable, whereas 17.19 × 10^4^ km^2^ were lost and only 0.51 × 10^4^ km^2^ were gained. Under SSP3-7.0, habitat loss increased to 14.91 × 10^4^ km^2^, while habitat expansion reached 1.7.0 × 10^4^ km^2^. SSP5-8.5 showed the greatest habitat expansion (8.13 × 10^4^ km^2^), the smallest habitat loss (13.23 × 10^4^ km^2^), and the largest stable area (10.69 × 10^4^ km^2^). Compared with the current habitat, the total suitable habitat area was projected to decrease by 69.78% under SSP1-2.6 and 55.26% under SSP3-7.0, and 21.32% under SSP5-8.5. Thus, although suitable habitat was projected to decline under all three future climate scenarios, SSP5-8.5 retained the largest stable habitat area, exhibited the greatest habitat expansion, and showed the smallest overall reduction in suitable habitat. Overall, SSP5-8.5 was associated with greater habitat persistence and expansion than SSP1-2.6 and SSP3-7.0.

**Figure 12:**
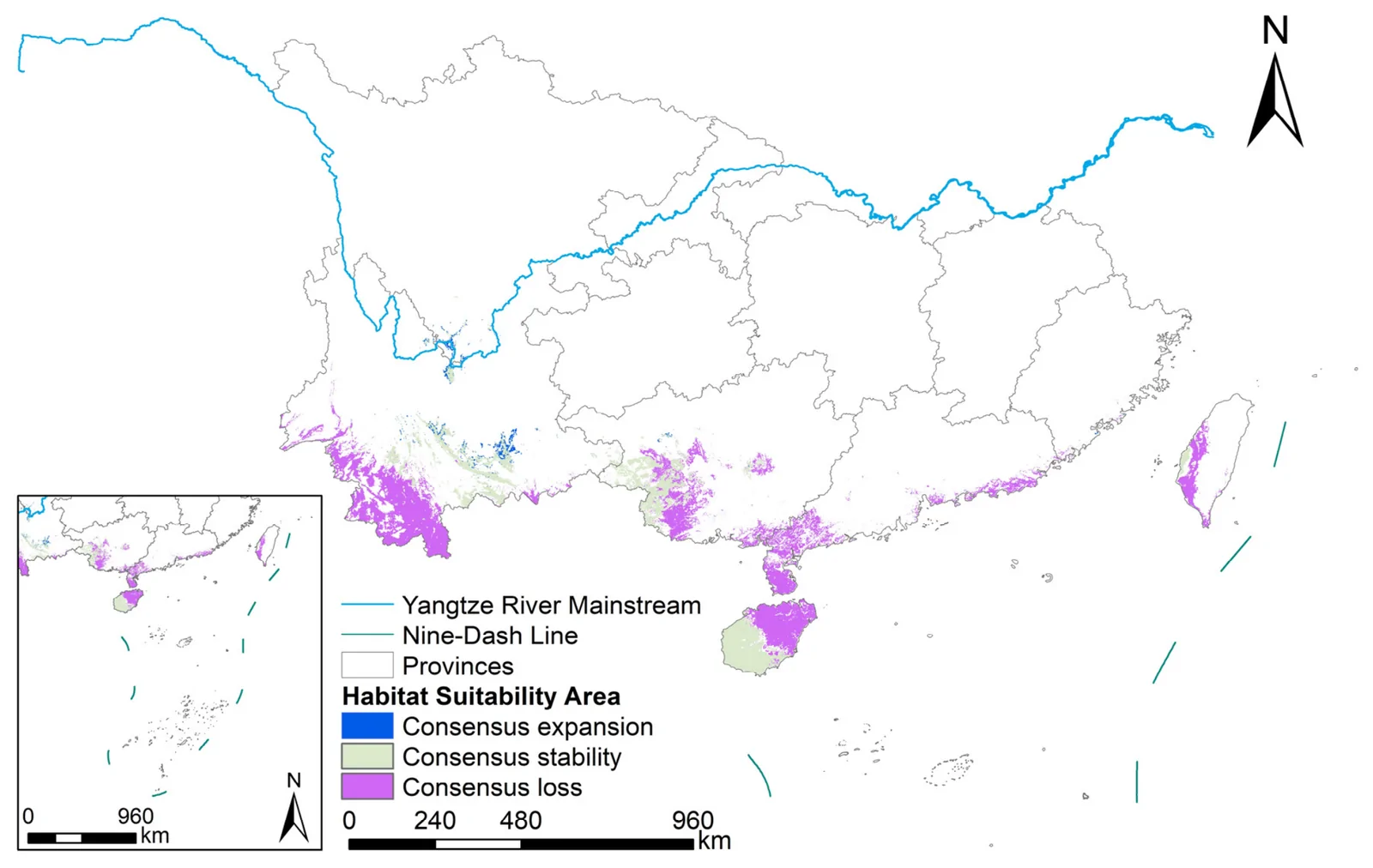
Consensus map of habitat expansion, stability and loss for *D. cochinchinensis* under SSP1-2.6, SSP3-7.0 and SSP5-8.5 scenarios in the 2090s relative to the current distribution.

As shown in Figure 12, consensus analysis across the three future climate scenarios revealed clear patterns of habitat change relative to the current suitable habitat in the 2090s. Consensus stable habitats were mainly distributed in the central and southwestern parts of Hainan Island, south-central Yunnan, and western Guangxi. Consensus habitat loss was more extensive and was concentrated in southwestern and southern Yunnan, southern Guangxi, the coastal areas of Guangdong and Fujian, and western Taiwan, with substantial losses also occurring in northern and eastern Hainan Island. In contrast, consensus habitat expansion was limited and occurred only as scattered patches in north-central Yunnan and west-central Guangxi.

**Figure 13:**
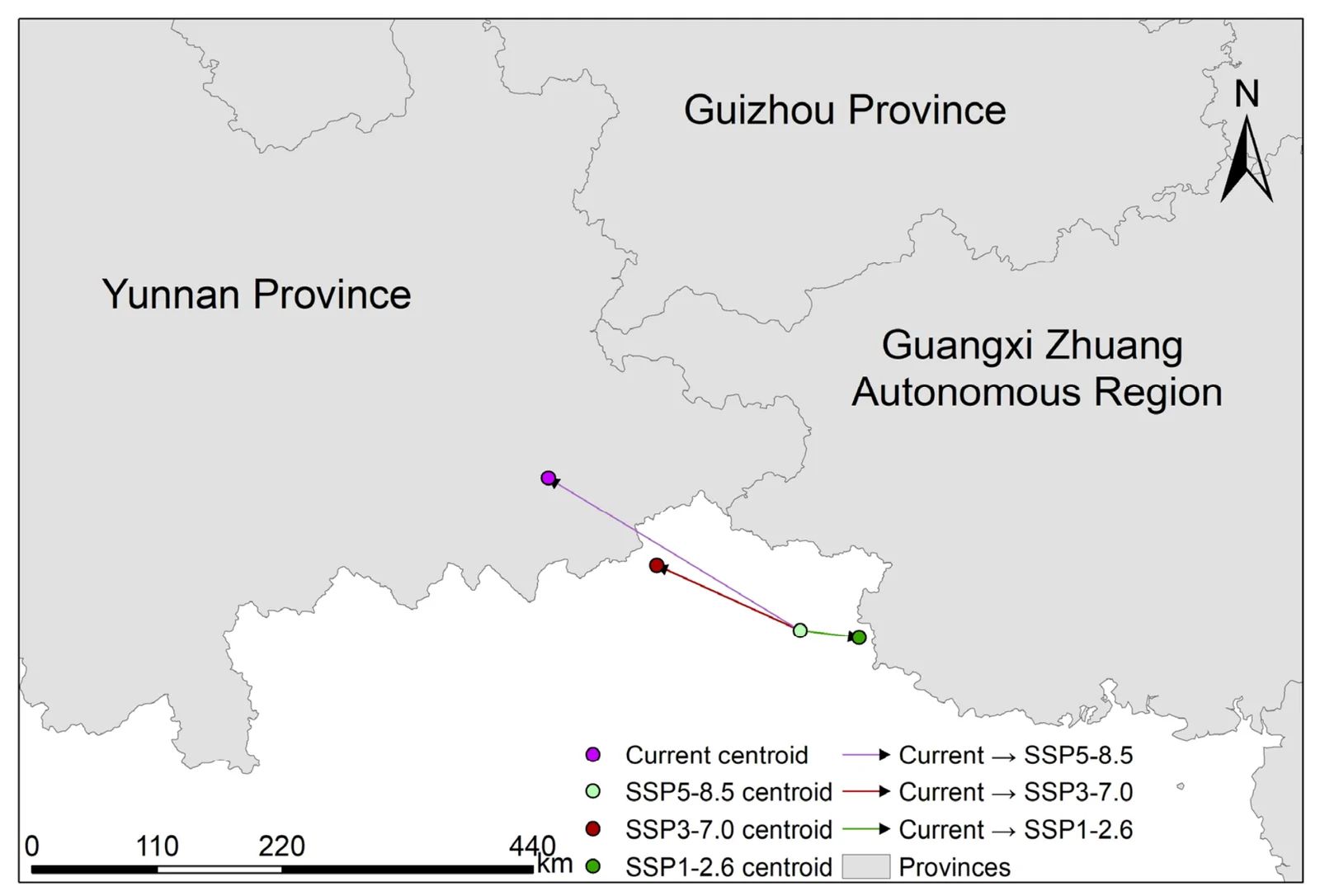
Centroid shifts in suitable habitats for *D. cochinchinensis* under future climate scenarios (SSP1-2.6, SSP3-7.0, and SSP5-8.5) in the 2090s relative to the current distribution.

The centroid of the current suitable habitat of *D. cochinchinensis* was located in southwestern Guangxi Zhuang Autonomous Region (106.11° E, 22.28° N) (Figure 13). Under future climate scenarios, the centroid exhibited different migration patterns. Under SSP1-2.6, the centroid shifted eastward to 106.57° E, 22.22° N, corresponding to a migration distance of 48.94 km and remaining within the Guangxi Zhuang Autonomous Region. Under SSP3-7.0, the centroid moved northwestward to 104.97° E, 22.79° N, with a migration distance of 129.84 km. Under SSP5-8.5, the centroid further moved northwestward to 104.12° E, 23.48° N in southeastern Yunnan Province, with the largest migration distance of 244.07 km. Overall, the magnitude of centroid displacement increased substantially from SSP1-2.6 to SSP5-8.5, with the centroid shifting progressively northwestward toward southeastern Yunnan under SSP3-7.0 and SSP5-8.5.

## 4. Discussion

### 4.1. Model Reliability and Environmental Determinants of Habitat Suitability

The optimized MaxEnt model showed excellent predictive performance, with a mean AUC of 0.960 ± 0.016, an avg.diff.AUC of 0.015, and a CBI value of 0.961. These evaluation metrics demonstrate that the optimized model was robust and reliable for predicting habitat suitability of *D. cochinchinensis*. Previous studies have demonstrated the utility of MaxEnt for predicting suitable habitats of endangered, medicinal, and economically important plant species under current and future climate conditions [51,61,62]. However, model performance metrics alone do not fully capture ecological realism. Therefore, model performance should be evaluated alongside response curves, variable importance, species ecology, and field distribution records.

Climate-related variables were the dominant model predictors of the habitat suitability of *D. cochinchinensis*, with Bio9, Bio7, and Bio15 showing the strongest associations with predicted suitability (Figure 3 and Figure 4). This pattern is consistent with the ecology of *D. cochinchinensis*, a tropical to subtropical tree species mainly in southwestern Guangxi and southern Yunnan near the northern margin of tropical Asia [3,63]. Its broad-scale distribution is therefore likely constrained primarily by climatic suitability, particularly thermal conditions, and subsequently modified by edaphic and topographic factors. Similar patterns have been reported for other warm-adapted plants in China. For example, MaxEnt modeling of *Zanthoxylum armatum* showed that its suitable habitats were concentrated in subtropical regions and were strongly associated with temperature-related factors [64]. The distribution of the near-threatened rosewood *Dalbergia cultrata* Graham ex Benth., which occurs across the Indo-China Peninsula and southern Yunnan, was also strongly influenced by annual temperature range [65]. Likewise, temperature annual range and minimum temperature of the coldest month were important predictors of *Litsea cubeba* distribution in China [66]. Collectively, these findings indicate that thermal conditions play a central role in shaping the distribution limits of warm-adapted woody plants.

Among the selected variables, Bio9 had the highest percent contribution and showed the strongest independent predictive ability in the jackknife test (Figure 4). The response curve indicated that dry-season temperature is an important climatic factor associated with the distribution of *D. cochinchinensis*. Although southwestern China is generally characterized by humid subtropical and tropical monsoon climates, relatively lower temperatures during the driest quarter may reduce climatic suitability for this warm-adapted species at the northern margin of tropical Asia. Similar climatic constraints have been reported for the endangered medicinal plant *H. riparia* in Yunnan [67]. Therefore, the high importance of Bio9 likely reflects the sensitivity of *D. cochinchinensis* to dry-season thermal limitation. Bio7 (annual temperature range) and Bio15 (precipitation seasonality) further highlighted the combined role of thermal variability and seasonal moisture regimes in shaping habitat suitability. Bio7 reflects annual thermal variability, and greater temperature fluctuations may impose additional stress on this warm-adapted species. Similar patterns have been reported for *Dalbergia cultrata* and *Dendrocalamus sinicus*, for which Bio7 was identified as a key determinant of habitat suitability [65,68]. In contrast, Bio15 represents the degree of seasonal variation in precipitation, suggesting that the distribution of *D. cochinchinensis* is also associated with the seasonal allocation and variability of water availability. Such precipitation seasonality may influence plant growth and population persistence by altering the temporal balance between wet- and dry-season moisture conditions. Overall, Bio9, Bio7, and Bio15 represent complementary aspects of climatic conditions, including dry-season temperature, growing-season warmth, and precipitation seasonality. Their combined influence suggests that the distribution of *D. cochinchinensis* is primarily shaped by the interactive effects of thermal conditions and seasonal moisture regimes rather than by a single climatic factor.

Lith and ST also contributed substantially to the model among the non-climatic variables, indicating that substrate and edaphic conditions may influence habitat suitability [51,69]. The response patterns showed that suitable habitats were associated with slate, phyllite/schist, limestone and other carbonate rocks, bare rock/rock outcrops, and diorite. suggesting that the species is associated with multiple substrate types rather than exclusively with limestone at the regional scale [70]. Notably, the relatively high suitability associated with limestone and exposed rock is consistent with the species’ frequently reported occurrence in limestone and karst habitats [71,72,73]. Similarly, Haplic Ferralsols, Ferric Acrisols, and Haplic Luvisols showed relatively high fitted suitability, although these soil-specific responses should not be interpreted as direct edaphic preferences. Therefore, Lith and ST likely represent important environmental correlates of *D. cochinchinensis* distribution rather than independent determinants of habitat suitability. Karst microhabitats and exposed rock have been shown to influence vegetation structure and habitat heterogeneity in limestone ecosystems [74], highlighting the potential ecological relevance of substrate-related factors. Future studies incorporating explicit karst distribution or carbonate geology layers may further clarify the relationship between *D. cochinchinensis* and karst environments.

### 4.2. Spatial Redistribution of Suitable Habitats

The current distribution pattern predicted by the optimized MaxEnt model is consistent with the affinity of *D. cochinchinensis* for tropical and subtropical environments. Suitable habitats were concentrated in southern China, particularly in Yunnan, Guangxi, Hainan, Taiwan, Guangdong, and Fujian. This pattern agrees with the known distribution of *D. cochinchinensis*, which occurs mainly in southern Yunnan and Guangxi and neighboring regions of tropical Asia [3]. Although the predicted suitable areas extended beyond the currently documented natural distribution of the species, this discrepancy is expected because SDMs estimate potential habitat suitability rather than realized distributions. The occurrence of *D. cochinchinensis* may also be limited by limestone substrate availability, cliff microhabitats, dispersal constraints, interspecific competition, land-use change, and long-term human disturbance. Therefore, model-predicted suitable areas in Hainan, Taiwan, Guangdong, and Fujian should be regarded as potential climate analogues or priority regions for field validation rather than confirmed natural distribution areas [75,76].

Future projections revealed marked differences among climate scenarios. Suitable habitat declined substantially under all three future scenarios, with the greatest reduction under SSP1-2.6, followed by SSP3-7.0, whereas SSP5-8.5 showed the smallest overall reduction and retained the largest stable and expansion areas. This pattern may reflect the thermophilic nature of *D. cochinchinensis*. Under the SSP5-8.5 scenario, stronger warming may make some currently cool inland and higher-latitude regions more thermally suitable for the species, thereby increasing the extent of climatically suitable habitats. Nevertheless, the total suitable habitat under SSP5-8.5 remained smaller than that under the current conditions, indicating that such expansion was insufficient to fully offset habitat loss. Consistency analysis further indicated that the total suitable habitat under SSP5-8.5 remained smaller than that under the current conditions, indicating that such expansion was insufficient to fully offset habitat loss (Figure 12). These climatically stable areas may serve as potential habitats for the long-term persistence of *D. cochinchinensis* under future climate change.

Centroid shifts were scenario dependent, with a slight eastward shift under SSP1-2.6 but pronounced northwestward shifts under SSP3-7.0 and SSP5-8.5, suggesting an increasing tendency for suitable habitats to redistribute toward inland mountainous regions under stronger climate warming. This pattern is partly consistent with the tendency of many plant species to track suitable climates toward higher latitudes or cooler mountainous regions under climate warming [77,78,79]. Southeastern Yunnan and adjacent mountainous areas may therefore provide important future habitats for *D. cochinchinensis*, particularly where warm dry-season temperatures, suitable precipitation, limestone substrates, and low anthropogenic disturbance coincide. From a conservation perspective, climatically stable habitats identified across all future climate scenarios, particularly those in southern and central Hainan, southwestern Guangxi, south-central Yunnan, and the western coastal region of Taiwan, should be regarded as priority conservation areas because they are most likely to represent long-term candidate stable habitats for *D. cochinchinensis*. Areas projected to become suitable under future climates may serve as potential targets for habitat restoration, long-term ecological monitoring, and assisted migration where appropriate. In contrast, regions consistently predicted to lose suitability across all scenarios should receive priority attention for population monitoring, germplasm collection, and ex situ conservation to minimize the loss of existing genetic resources.

### 4.3. Limitations and Future Work

Although the optimized MaxEnt model provided a useful framework for predicting the current and future habitat suitability of *D. cochinchinensis*, several limitations should be considered. First, presence-only models estimate potential environmental suitability rather than realized species distributions, and predictions may be influenced by occurrence-data bias, study-area extent, predictor selection, spatial resolution, and parameter settings [80,81,82]. Second, independent field validation and additional occurrence data are still needed to further assess model transferability and reliability. Third, the model primarily incorporated macroclimatic, topographic, lithology, soil, and land-use variables, whereas fine-scale ecological factors, including microhabitats, interspecific competition, dispersal limitation, and human disturbance, were not fully represented [82,83]. In addition, variable spatial resolution among environmental datasets and uncertainties from resampling and scaling may bias model outputs. Future modelling can be improved by adopting high-resolution detailed environmental data. Future habitat projections were generated using a single CMIP6 climate model (BCC-CSM2-MR) and should therefore be interpreted as exploratory assessments rather than definitive predictions. Moreover, future projections may involve additional uncertainties associated with extrapolation into non-analogue environmental conditions, suitability threshold selection, and interactions between climate change and future land-use dynamics. Extrapolation diagnostics, such as clamping maps and Multivariate Environmental Similarity Surface (MESS) analyses, were not performed in this study but would be valuable for identifying areas beyond the environmental conditions represented in the calibration data and improving confidence in future projections. Future studies incorporating multi-model CMIP6 ensembles, together with high-resolution environmental data and field-based ecological information, would help reduce uncertainties and improve the robustness of future projections. Consequently, newly suitable areas should be regarded as candidate regions for further investigation rather than confirmed future habitats. Combining species distribution modeling with long-term monitoring, demographic information, genetic diversity assessments, and conservation experiments may help identify stable refugia, evaluate the feasibility of assisted migration, and support more effective conservation planning for this nationally protected medicinal species under climate and land-use change.

## 5. Conclusions

This study evaluated the current and future suitable habitats of *D. cochinchinensis* in China using an optimized MaxEnt model. The model demonstrated high predictive performance and identified climatic variables as the dominant model predictors of habitat suitability, particularly the mean temperature of the driest quarter (Bio9), annual temperature range (Bio7), and precipitation seasonality (Bio15). Lithology (Lith) and soil type (ST) were the major non-climatic contributors, whereas Land use/land cover (LULC) showed comparatively limited importance in the full model. Under current climatic conditions, suitable habitats covered 23.93 × 10^4^ km^2^ (8.31% of the study area) and were concentrated in southern Yunnan, Guangxi, Guangdong, Hainan, Taiwan, and scattered parts of Fujian. Scenario-based projections under BCC-CSM2-MR indicated marked changes in both habitat extent and spatial distribution. Suitable habitat area decreased under all three future scenarios, by 69.78% under SSP1-2.6, 55.26% under SSP3-7.0, and 21.32% under SSP5-8.5. Despite the overall contraction, SSP5-8.5 retained the largest stable habitat area and showed the greatest habitat expansion among the three scenarios. The spatial comparison among the three future climate scenarios revealed consistent patterns of habitat persistence, loss, and expansion. Stable habitats were mainly distributed in south-central Yunnan, western Guangxi, and central and southwestern Hainan, whereas habitat losses were concentrated in southwestern Yunnan, southern Guangxi, coastal Guangdong and Fujian, western Taiwan, and northern and eastern Hainan. Habitat expansion was limited and occurred mainly as scattered patches in north-central Yunnan and west-central Guangxi. Habitat centroid shifts were scenario dependent, with an eastward shift of 48.94 km under SSP1-2.6 and northwestward shifts of 129.84 and 244.07 km under SSP3-7.0 and SSP5-8.5, respectively. Although the future projections incorporated both climate and LULC changes, these results indicate that future climate change may be the primary factor substantially altering the distribution of suitable habitats for *D. cochinchinensis*, particularly under medium- and high-emission scenarios.

## Data Availability

The occurrence records of *D. cochinchinensis* used in this study were compiled from publicly available sources, including the Global Biodiversity Information Facility (GBIF; https://www.gbif.org/) (GBIF.org, GBIF Occurrence Download. Available online: https://doi.org/10.15468/dL.k42bkq (accessed on 24 November 2024)), the Chinese Virtual Herbarium (CVH; https://www.cvh.ac.cn/), the National Specimen Information Infrastructure (NSII; http://www.nsii.org.cn/), Flora of China (http://www.iplant.cn/foc/) (accessed on 24 November 2024), and published literature. Environmental variables were obtained from publicly accessible databases, including WorldClim v2.1 (https://www.worldclim.org/), the Harmonized World Soil Database (HWSD v1.2; https://www.fao.org/soils-portal/data-hub/soil-maps-and-databases/harmonized-world-soil-database-v12/en/) (accessed on 24 November 2024), the lithology dataset from the Soil and Terrain Database (SOTER; https://isric.org/explore/soter) (accessed on 7 July 2026) developed by the Food and Agriculture Organization (FAO) and the International Soil Reference and Information Centre (ISRIC), current LULC data from the 30 m annual China Land Cover Dataset (CLCD; https://zenodo.org/records/18180184) (accessed on 24 November 2024), and the future land-use/land-cover dataset developed by Zhang et al. (2023) [47] (https://doi.org/10.6084/m9.figshare.23542860) (accessed on 24 November 2024). The processed datasets and model outputs are available from the corresponding author upon reasonable request. To protect this nationally protected species, precise occurrence coordinates are not publicly released.

## Supplementary Materials

The following supporting information can be downloaded at: https://www.mdpi.com/article/10.3390/plants15162539/s1. Table S1. Software environment, model settings, and optimization criteria used for MaxEnt model tuning. Table S2. Evaluation metrics of the optimal MaxEnt parameter combination identified by ENMeval. Table S3. Codes and corresponding classifications of categorical environmental variables used in the MaxEnt model for *D. cochinchinensis*. Table S4. Centroid coordinates and migration distances of suitable habitat for *D. cochinchinensis* under current and future climate scenarios. Figure S1. Evaluation of MaxEnt feature-class and regularization-multiplier combinations based on AICc, ΔAICc, avg.OR10, and avg.diff.AUC.

## Abbreviations

The following abbreviations are used in this manuscript:

AUC	Area Under the Receiver Operating Characteristic Curve
Bio	Bioclimatic variable
CMIP6	Coupled Model Intercomparison Project Phase 6
ENMeval	MaxEnt model evaluation and tuning package
FC	Feature Class
GBIF	Global Biodiversity Information Facility
GCAM	Global Change Analysis Model
GIS	Geographic Information System
HWSD	Harmonized World Soil Database
IUCN	International Union for Conservation of Nature
LULC	Land Use/Land Cover
MaxEnt	Maximum Entropy Model;
PLUS	Patch-generating Land Use Simulation
RCP	Representative Concentration Pathway
RM	Regularization Multiplier
SDM	Species Distribution Model
SLP	Slope
SSP	Shared Socioeconomic Pathway
Lith	Lithology
ST	Soil Type
T_pH_H2O	Soil pH measured in water
VIF	Variance Inflation Factor

## Appendix A

**Table A1:** Summary of environmental variables used for modeling the distribution of *D. cochinchinensis* in this study.

Category	Abbreviation	Description	Unit	Source	Original Resolution
**Climate**	Bio1	Annual mean temperature	°C	WorldClim 2.1	~1 km
	Bio2	Mean diurnal range (mean of monthly (max temp-min temp))	°C		
	Bio3	Isothermality (Bio2/Bio7) (×100)	%		
	Bio4	Temperature seasonality (standard deviation × 100)	-		
	Bio5	Max temperature of warmest month	°C		
	Bio6	Min temperature of coldest month	°C		
	Bio7	Temperature annual range (Bio5-Bio6)	°C		
	Bio8	Mean temperature of wettest quarter	°C		
	Bio9	Mean temperature of driest quarter	°C		
	Bio10	Mean temperature of warmest quarter	°C		
	Bio11	Mean temperature of coldest quarter	°C		
	Bio12	Annual precipitation	mm		
	Bio13	Precipitation of wettest month	mm		
	Bio14	Precipitation of driest month	mm		
	Bio15	Precipitation seasonality (Coefficient of variation)	-		
	Bio16	Precipitation of wettest quarter	mm		
	Bio17	Precipitation of driest quarter	mm		
	Bio18	Precipitation of warmest quarter	mm		
	Bio19	Precipitation of coldest quarter	mm		
**Lithology**	Lith	Lithological characteristics	-	SOTER	1:1,000,000 scale
**Soil**	ST	Soil type	-	HWSD	1 km
	T_PH_H2O	Soil pH in water for the depth of top layer	−log(H+)		
**Anthropogenic**	LULC	Land use/Land cover	-	ESA-CCI (2000; Current scenario) [46];	300 m
				Zhang et al. (2023) (2090s; Future scenarios) [47]	1 km
**Topography**	Elev	Elevation	m	WorldClim	~1 km
	SLP	Slope	°		
	Northness	Northness	-	Derived from Aspect (WorldClim)	
	Eastness	Eastness	-		
