# Supplementary material for: Habitat Redistribution of Dracaena cochinchinensis (Lour.) S.C.Chen in Southern China Under Climate and Land-Use Change: Current Distribution, Future Projections, and Conservation Implications
Source: Plants (Basel). 2026 Aug 21;15(16):2539. doi: 10.3390/plants15162539 (PMC13516889; doi:10.3390/plants15162539)
Supplement: Supplementary file 1 [file plants-15-02539-s001.zip › plants-4402699-supplementary.pdf]

Supplementary materials

Table S1. Software environment, model settings, and optimization criteria used for MaxEnt model tuning.

Item	Setting / Version
ENMeval version	ENMeval version 2.0.5.2
MaxEnt version	3.4.4
Java version	1.8.0_481
Partitioning method	Block spatial partitioning
Number of spatial partitions	4
MaxEnt output type	Logistic
Number of RM–FC combinations	240
Model-selection criterion	Minimum AICc
ΔAICc of selected model	0
Extrapolation	Enabled
Clamping	Enabled
Random seed	Not explicitly specified
Active/non-zero coefficients	8–187 across the 240 candidate models

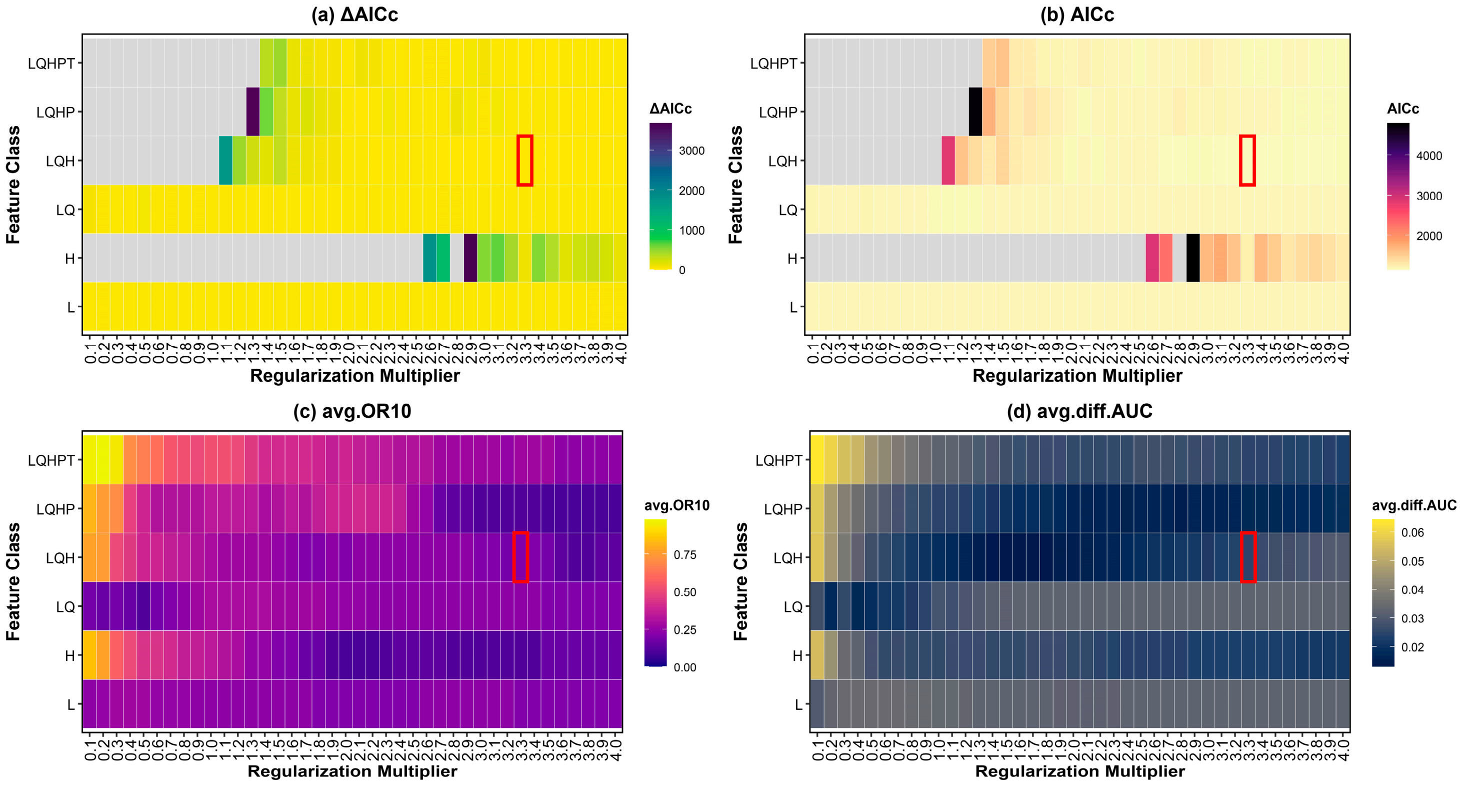

Figure S1. Evaluation of MaxEnt feature-class and regularization-multiplier combinations based on AICc, ΔAICc, avg.OR10, and avg.diff.AUC. The red box indicates the optimal parameter combination (FC = LQH, RM = 3.3). Gray cells in panels (a) and (b) represent parameter combinations for which AICc-related values were unavailable (NA).

Table S2. Evaluation metrics of the optimal MaxEnt parameter combination identified by ENMeval.

FC	RM	avg.diff.AUC	avg.OR10	AICc	delta.AICc
LQH	3.3	0.0248	0.2045	1131.550	0
LQ	1.2	0.0291	0.2955	1155.479	0

Table S3. Codes and corresponding classifications of categorical environmental variables used in the MaxEnt model for *D. cochinchinensis*.

(a) Soil type (ST)

Value	Code	FAO90_name	Major group
0	—	Not covered / unmapped in source soil databases	Miscellaneous / unmapped
2	FLe	Eutric Fluvisols	Fluvisols
3	FLc	Calcaric Fluvisols	Fluvisols
10	GLe	Eutric Gleysols	Gleysols
11	GLk	Calcic Gleysols	Gleysols
14	GLm	Mollic Gleysols	Gleysols
16	GLt	Thionic Gleysols	Gleysols
17	GLi	Gelic Gleysols	Gleysols
19	ACh	Haplic Acrisols	Acrisols
20	ACf	Ferric Acrisols	Acrisols
21	ACu	Humic Acrisols	Acrisols
22	ACp	Plinthic Acrisols	Acrisols
25	ALh	Haplic Alisols	Alisols
26	ALf	Ferric Alisols	Alisols
29	ALj	Stagnic Alisols	Alisols
32	ANh	Haplic Andosols	Andosols
34	ANu	Umbric Andosols	Andosols
39	ARh	Haplic Arenosols	Arenosols
44	ARc	Calcaric Arenosols	Arenosols
48	ATc	Cumulic Anthrosols	Anthrosols
53	CHk	Calcic Chernozems	Chernozems
54	CHl	Luvic Chernozems	Chernozems
58	CLh	Haplic Calcisols	Calcisols
62	CMe	Eutric Cambisols	Cambisols
63	CMd	Dystric Cambisols	Cambisols
64	CMu	Humic Cambisols	Cambisols
65	CMc	Calcaric Cambisols	Cambisols
66	CMx	Chromic Cambisols	Cambisols
68	CMc	Ferralic Cambisols	Cambisols
70	CMi	Gelic Cambisols	Cambisols
72	FRh	Haplic Ferralsols	Ferralsols
73	FRx	Xanthic Ferralsols	Ferralsols
79	GRh	Haplic Greyzems	Greyzems
88	HSs	Terric Histosols	Histosols
89	HSf	Fibric Histosols	Histosols
97	LP	Leptosols	Leptosols

98	LPe	Eutric Leptosols	Leptosols
99	LPd	Dystric Leptosols	Leptosols
100	LPk	Rendzic Leptosols	Leptosols
101	LPm	Mollic Leptosols	Leptosols
104	LPi	Gelic Leptosols	Leptosols
106	LVh	Haplic Luvisols	Luvisols
108	LVx	Chromic Luvisols	Luvisols
109	LVk	Calcic Luvisols	Luvisols
111	LVa	Albic Luvisols	Luvisols
113	LVg	Gleyic Luvisols	Luvisols
116	LXf	Ferric Lixisols	Lixisols
118	LXa	Albic Lixisols	Lixisols
124	NTu	Humic Nitisols	Nitisols
127	PDd	Dystric Podzoluvisols	Podzoluvisols
132	PHh	Haplic Phaeozems	Phaeozems
133	PHc	Calcaric Phaeozems	Phaeozems
138	PLe	Eutric Planosols	Planosols
156	RGe	Eutric Regosols	Regosols
157	RGc	Calcaric Regosols	Regosols
159	RGd	Dystric Regosols	Regosols
163	SCh	Haplic Solonchaks	Solonchaks
165	SCg	Gleyic Solonchaks	Solonchaks
178	VRe	Eutric Vertisols	Vertisols
194	DS	Dunes and shifting sands	Miscellaneous / non-soil unit
196	RK	Rock debris / bare rock	Miscellaneous / non-soil unit
198	WR	Inland/Open water	Miscellaneous / non-soil unit
199	GG	Glaciers and permanent snow	Miscellaneous / non-soil unit
200	ND	No data	Miscellaneous / non-soil unit
201	UR	Urban	Miscellaneous / non-soil unit
204	FP	Fishponds	Miscellaneous / non-soil unit

(b) Lithology (Lith)

Value	Code	Major class	Primary_meaning
1	UF	Unconsolidated material	Fluvial deposits
2	IA1	Igneous rock	Diorite
3	UL	Unconsolidated material	Lacustrine deposits
4	UG	Unconsolidated material	Glacial deposits
5	MA2	Metamorphic rock	Gneiss, migmatite
6	UE1	Unconsolidated material	Loess
7	SC2	Sedimentary rock	Sandstone, greywacke, arkose
8	IP4	Igneous / pyroclastic material	Ignimbrite
9	II1	Igneous rock	Andesite, trachyte, phonolite
10	SO1	Sedimentary rock	Limestone and other carbonate rocks
11	SC4	Sedimentary rock	Shale
12	UE2	Unconsolidated material	Eolian sand / sand
13	WR	Miscellaneous	Water

14	GG	Miscellaneous	Glacier / permanent ice and snow
15	MB1	Metamorphic rock	Slate, phyllite (pelitic rocks)
16	UF2	Unconsolidated material	Fluvial clay, silt and loam
17	IB2	Igneous rock	Basalt
18	IP2	Igneous / pyroclastic material	Volcanic scoria / breccia
19	UO	Unconsolidated material	Organic deposits
20	UF/UL	Unconsolidated material	Composite: Fluvial deposits / Lacustrine deposits
22	SC3	Sedimentary rock	Siltstone, mudstone, claystone
25	SC1	Sedimentary rock	Conglomerate, breccia
26	MA3/MB1	Metamorphic rock	Composite: — / Slate, phyllite (pelitic rocks)
28	UM	Unconsolidated material	Marine / estuarine deposits
30	UR	Unconsolidated / residual material	Weathered residuum
31	SC	Sedimentary rock	Clastic sedimentary rocks (group-level code)
32	UR1	Unconsolidated / residual material	Bauxite, laterite
34	SO2	Sedimentary rock	Marl and other mixtures
35	MA4/MB2		Composite: — / —
37	SC2/SC4	Sedimentary rock	Composite: Sandstone, greywacke, arkose / Shale
41	RK	Miscellaneous	Bare rock / rock outcrop
43	MB1/MB2	Metamorphic rock	Composite: Slate, phyllite (pelitic rocks) / —
47	SC7	Sedimentary rock (consolidated)	Source-specific subtype; exact lithology unresolved
48	UF/UM	Unconsolidated material	Composite: Fluvial deposits / Marine / estuarine deposits
49	MA1	Metamorphic rock	Quartzite
51	UM/UF	Unconsolidated material	Composite: Marine / estuarine deposits / Fluvial deposits
52	SC5	Sedimentary rock	Ironstone
53	IA2	Igneous rock	Granodiorite
54	UL/UM	Unconsolidated material	Composite: Lacustrine deposits / Marine / estuarine deposits
55	SC16	Sedimentary rock (consolidated)	Source-specific subtype; exact lithology unresolved
56	IA4	Igneous rock	Rhyolite

(c) Land-use/land-cover (LULC)

Value	Class
1	Cropland
2	Forest
3	Grassland
4	Urban
5	Barren land
6	Water

Table S4. Centroid coordinates and migration distances of suitable habitat for *D. cochinchinensis* under current and future climate scenarios.

**Scenario**	**Longitude (°E)**	**Latitude (°N)**	**Migration distance from current centroid (km)**
Current	106.11	22.28	0
SSP1-2.6 (2090s)	106.57	22.22	48.94
SSP3-7.0 (2090s)	104.97	22.79	129.84
SSP5-8.5 (2090s)	104.12	23.48	244.07

Note: Centroids were calculated from the spatial distribution of predicted suitable habitat under each scenario. Migration distance represents the straight-line distance between the centroid under each future scenario and the centroid under current conditions
